# Genotyping by Sequencing for SNP-Based Linkage Analysis and Identification of QTLs Linked to Fruit Quality Traits in Japanese Plum (*Prunus salicina* Lindl.)

**DOI:** 10.3389/fpls.2017.00476

**Published:** 2017-04-11

**Authors:** Juan A. Salazar, Igor Pacheco, Paulina Shinya, Patricio Zapata, Claudia Silva, Mallikarjuna Aradhya, Dianne Velasco, David Ruiz, Pedro Martínez-Gómez, Rodrigo Infante

**Affiliations:** ^1^Departamento de Producción Agrícola, Universidad de ChileSantiago, Chile; ^2^Laboratorio de Bioinformática y Expresión Génica, Instituto de Nutrición y Tecnología de Alimentos, Universidad de ChileSantiago, Chile; ^3^National Clonal Germplasm Repository, ARS, USDADavis, CA, USA; ^4^Departamento de Mejora Vegetal, CEBAS-CSICMurcia, Spain

**Keywords:** Japanese plum, breeding, fruit quality, molecular markers, ripening, *Prunus salicina*, SNP, GBS

## Abstract

Marker-assisted selection (MAS) in stone fruit (*Prunus* species) breeding is currently difficult to achieve due to the polygenic nature of the most relevant agronomic traits linked to fruit quality. Genotyping by sequencing (GBS), however, provides a large quantity of useful data suitable for fine mapping using Single Nucleotide Polymorphisms (SNPs) from a reference genome. In this study, GBS was used to genotype 272 seedlings of three F1 Japanese plum (*Prunus salicina* Lindl) progenies derived from crossing “98–99” (as a common female parent) with “Angeleno,” “September King,” and “September Queen” as male parents. Raw sequences were aligned to the Peach genome v1, and 42,909 filtered SNPs were obtained after sequence alignment. In addition, 153 seedlings from the “98–99” × “Angeleno” cross were used to develop a genetic map for each parent. A total of 981 SNPs were mapped (479 for “98–99” and 502 for “Angeleno”), covering a genetic distance of 688.8 and 647.03 cM, respectively. Fifty five seedlings from this progeny were phenotyped for different fruit quality traits including ripening time, fruit weight, fruit shape, chlorophyll index, skin color, flesh color, over color, firmness, and soluble solids content in the years 2015 and 2016. Linkage-based QTL analysis allowed the identification of genomic regions significantly associated with ripening time (LG4 of both parents and both phenotyping years), fruit skin color (LG3 and LG4 of both parents and both years), chlorophyll degradation index (LG3 of both parents in 2015) and fruit weight (LG7 of both parents in 2016). These results represent a promising situation for GBS in the identification of SNP variants associated to fruit quality traits, potentially applicable in breeding programs through MAS, in a highly heterozygous crop species such as Japanese plum.

## Introduction

Japanese plum (*Prunus salicina* Lindl.) is a crop tree species native to China. It has a diploid genome (2*n* = 2*x* = 16), unlike the European plum (*P. domestica* L.), which has a hexaploid genome (6*n* = 2*x* = 48), although both species belong to the *Rosaceae* family, subfamily *Prunoideae*, genus *Prunus, and* subgenus *Prunophora* (Topp et al., 2012). Plums, including Japanese plum and European plum, with a production of 11.52 million tons (Mt) in 2015, are the second stone fruit production after peaches and nectarines (annual production of 21.63 Mt). China is by far the world leading producer of plums (5.90 Mt) based on Japanese plums. Another major Japanese plum-producing area includes the European countries bordering the Mediterranean Sea, mainly Spain (0.21 Mt), France (0.20 Mt), and Italy (0.19 Mt). USA is another major plum producer (0.24 Mt) for European and Japanese plums, and the most important exporting country of dried plums. In the Southern hemisphere plum production is dominated by Chile (0.30 Mt) followed by Argentina (0.15 Mt), also based on Japanese plum production (http://faostat.fao.org).

Most commercial Japanese plum production is, however, based on a few cultivars, and it is therefore necessary to diversify the range of varieties to accelerate breeding programs. The most advanced breeding programs of this species are in California (Okie and Ramming, 1999), although important programs are currently being developed in China, South Africa, Spain, Italy, Brazil, and Chile (Hartmann and Neumüller, 2009; Topp et al., 2012; Liu et al., 2013; Ruiz et al., 2016).

Marker-assisted selection (MAS) is an important biotechnological tool used in *Prunus* breeding programs to improve the efficiency of developing new genotypes, although its use is currently limited to traits with a low number of genes involved in their expression (Salazar et al., 2014). The polygenic nature of most of the useful traits (mainly related to fruit quality), with genes distributed throughout the entire genome, makes it very difficult to develop linked markers. Many authors have therefore focused on the study and characterization of these polygenic traits in different *Prunus* species from both a phenological point of view (Campoy et al., 2010; Dirlewanger et al., 2012; Socquet-Juglard et al., 2013; Castède et al., 2015; Eduardo et al., 2015; Nuñez-Lillo et al., 2015; Salazar et al., 2016), a pomological perspective (Sooriyapathirana et al., 2010; Zhang et al., 2010; Rosyara et al., 2013; Salazar et al., 2013; Da Silva Linge et al., 2015; Fresnedo-Ramírez et al., 2015, 2016; Lambert et al., 2016; Zeballos et al., 2016) and regarding pathogen resistance issues (Martínez-García et al., 2013a; Yang et al., 2013; Pacheco et al., 2014). These studies have been focused on the identification of QTLs in peach [*P. persica* (L.) Batsch] (2*n* = 2*x* = 16); sweet cherry (*P. avium* L.) (2*n* = 2*x* = 16); and apricot (*P. armeniaca* L.) (2*n* = 2*x* = 16).

In the case of Japanese plum, however, only a few researches have been focused to QTL identification. For example some studies are aimed for identifying QTLs linked to nematode [*Meloidogine incognita* Kofoid et White and *Meloidogine arenaria* (Neal) Chitwood] resistance using plum progenies and interspecific plum × almond [*P. amygdalus* (Batsch) syn. *P. dulcis* (Miller) Webb] (2*n* = 2*x* = 16) × peach progenies (Claverie et al., 2004; Dirlewanger et al., 2004a; Van Ghelder et al., 2010). As far as we know, the QTL analysis of traits linked to fruit quality has not yet been done in *P. salicina*. More studies focused on this species are therefore needed in order to incorporate MAS into the new breeding programs for *P. salicina* fruit quality.

In recent years it has become increasingly common to use SNP markers to get sutured genetic maps for greater accurate QTL identification using different techniques as Fresnedo-Ramírez et al. (2016) and Zeballos et al. (2016) which have been mapping 2,398 and 8,144 SNP markers respectively using IPSC 9K peach SNP array (Verde, 2012) identifying several QTLs for fruit quality traits in peach or Lambert et al. (2016) which identified major genes for fruit flesh color (Y), skin pubescence (G), stone adhesion-flesh texture (F-M), sub-acid fruit (D), and fruit shape (S) while Salazar et al. (2016) in apricot, using SNPlex technology identified significant QTLs for phenology traits especially for ripening time.

Lately, genotyping by sequencing (GBS) technology makes it possible to generate more saturated genetic maps, thus facilitating more accurate QTL identification. This next generation technology makes it possible to build libraries of thousands of DNA fragments (tags), as has been done in maize (IBM) and barley (Oregon Wolfe Barley), for which 200,000 and 25,000 sequence tags, respectively, have been mapped. A single GBS experiment can provide 25,000 SNPs, which can be used for germplasm characterization, plant breeding progeny studies and QTL mapping (Bielenberg et al., 2015; Guajardo et al., 2015).

GBS technology provides large quantities of quality data that can be used for fine mapping, identification of SNP markers from a reference genome and localization of QTLs for certain quality traits of interest linked to specific genes. GBS is a high-throughput technique that has been widely applied in different species for multiple purposes, including for heritability studies (Ashraf et al., 2016); diversity analysis in maize and barley (Elshire et al., 2011); population structure evaluation in Cassava (Rabbi et al., 2015); and genetic structure analysis in Poaceae (McAllister and Miller, 2016). The technology has also been used for seed characterization in chickpea (Verma et al., 2015) and for the identification of QTLs linked to different traits like pre-harvest sprouting and rust resistance in wheat (Lin et al., 2015; Bajgain et al., 2016) and club root resistance in cabbage (Lee et al., 2015). In fruit species, GBS has been used to identify QTLs linked to blooming date and low temperature requirements in peach (Bielenberg et al., 2015). GBS does not enable us to generate molecular markers for MAS (Yang et al., 2016), but it does make it possible to more precisely localize QTLs in order to perform deeper sequencing and generate effective molecular tools for MAS. The GBS technology could therefore be particularly useful in species such as Japanese plum and for studying traits like fruit quality traits, i.e., in cases where the molecular information is limited.

The objective of this work was to generate a saturated genetic map in *P. salicina* and to carry out the QTL identification of several important fruit quality traits in order to locate major QTLs and associated markers to help develop MAS strategies in breeding programs.

## Materials and methods

### Plant materials

The plant material assayed included 11 Japanese plum cultivars and 3 F1 progenies. The F1 Japanese plum progenies (272 seedlings) were generated in the Rinconada de Maipu Experimental Station (Santiago, Chile) in 2011 by bumble bee pollination under a fine plastic mesh cage using a common female parent genotype “98–99” and three different pollen donors, including “September King,” “September Queen,” and “Angeleno.” The “98–99” genotype is a mid-early maturing selection with red skin and yellow flesh and with high organoleptic quality. “Angeleno” is a mid-late maturing cultivar with purple skin color and excellent postharvest performance, while “September King” and “September Queen” are both very late maturing genotypes.

### Fruit quality evaluation

Fifty-five seedlings from the “98–99” × “Angeleno” progeny were phenotyped in the years 2015 and 2016. Nine agronomic traits linked to fruit quality were evaluated including ripening time (RT), fruit weight (FW), shape (SHP), skin color (SKC), flesh color (FLSC), over color (OVC), chlorophyll index (I_AD_), firmness (FIRM), and the soluble solids concentration (SSC). RT was calculated in Julian days and FW in grams. SHP and fruit color (SKC, FLSC, and OVC) were visually determined and classified in numerical categories. SHP was defined according to the following scale: elongated (1), hearted (2), spherical (3), or oblate (4), while SKC was classified as green (1), yellow (2), red (3), purple (4), or black (5). FLSC was classified as white (1), green (2), yellow (3), or red (4). In addition, OVC was determined on a scale of 1–4, determined as percentages of color covering the fruit: 25% (1), 50% (2), 75% (3), and 100% (4). On the other hand, FIRM was quantified in Newton (N) by a texture analyzer (TA.XT plus, Texture Technologies, Hamilton, MA, USA) using a 7.9 mm diameter plunger, and SSC was determined using an ATAGO® hand-held refractometer calibrated as the percentage of sucrose at 20°C. I_AD_ chlorophyll content-related maturity index was measured by DA-meter (Gottardi et al., 2009; Infante et al., 2011a). All traits were evaluated at the harvest date, which was determined according to Contador et al. (2016), i.e., when I_AD_ was between 1 and 1.4 units and the texture close to 40 N. I_AD_, FIRM, and SSC were evaluated in two maturity states: at the harvest date (1, giving place to I_AD__1, FIRM_1 and SSS_1) and 1 week after harvest (2, giving place to I_AD__2, FIRM_2, and SSC_2).

### Fruit quality data analysis

Descriptive tables and histograms showing the distribution of the proportion of seedlings were generated for each trait and year. The normality of the data was analyzed using the $Shapiro-Wilks test, and traits showing normal distribution were analyzed by ANOVA, considering genotype and year as independent factors. Traits that did not meet the normality criteria were analyzed with the Kruskal-Wallis non-parametric test. In addition, correlations between and within traits for each year were analyzed using Pearson coefficients, and a Generalized Linear Mixed Model (GLMM) using Restricted Maximum Likehood (REML) was calculated for determining the genetic effect of each seedling by Best Linear Unbiased Predictors (BLUPs), considering the genotype factor as a random effect. All analyses and histograms were calculated and edited using INFOSTAT v16 software with the exception of the GLMM analysis, which was calculated using an interface between INFOSTAT and R.

### DNA extraction and genotyping by sequencing (GBS)

Young leaves for each individual were collected and lyophilized for DNA extraction, which was carried out according to the CTAB procedure described by Doyle and Doyle (1987). A total of 283 DNA samples (the 272 seedlings from the three F1 Japanese plum progenies derived from crossing “98–99” as a common female parent with “Angeleno,” “September King,” and “September Queen” as male parents; and the 11 Japanese plum cultivars) were sent to the Biotechnology Resource Center (BRC) at Cornell University (New York, USA). Here, Genotype by Sequencing (GBS) technology was used to generate DNA fragment libraries from each offspring and parent. The GBS analysis pipeline (Tassel v.3.0.166) was run on these samples, which were digested with the enzyme ApeKI. This enzyme is partially methylation sensitive and has been successfully used in maize (Elshire et al., 2011). The results include FASTQ files (raw sequences); TOPM files (tags on a physical map), which contain unique tag sequences of 64 bp that were present across all samples and tag alignment information; Hapmap files, which include the SNP-calling output from the GBS bioinformatics pipeline; and VCF files (an alternative format for holding SNPs). Three lanes, one lane per plate, were used to generate the FASTQ files. Failed samples (non-blank) were defined as those with <10% of the mean reads per sample coming from the lane on which they were sequenced. The Peach genome v1 was used as a reference genome (Verde et al., 2013). VCFtool v0.1.11 was used to calculate depth and missingness from the unfiltered VCF file. Genotypes were filtered to leave only those with a quality of 98 or higher (high confidence SNP calls) with VCFtools. In addition, biallelic SNPs were converted to the PLINK format. The remaining individuals/sites were filtered according to missingness and allele frequency.

### Genetic linkage analysis

A Z paternity test at *p* = 0.05 using SNPs from each parent was applied in order to discern the parentage of the 272 seedlings. A total of 161 individuals were obtained from “Angeleno,” 62 from “September King” and 15 from “September Queen,” and the genotype “98–99” was the common female. One hundred fifty-three seedlings from the “98–99” × “Angeleno” progeny were used for genetic mapping. Genotyping errors, under-calling homozygotes and heterozygotes and SNPs with over 10% missing data were eliminated prior to genetic mapping. A total of 14,697 SNPs were obtained, including common SNPs (hkxhk; 21%) and uncommon SNPs for “98–99” (lmxll; 26%) and “Angeleno” (nnxnp; 53%). Genetic linkage maps for each parent were constructed by JoinMap v.3.0 software (Van Ooijen, 2006) using the Kosambi mapping function with a frequency of recombination of 0.3, and a LOD value over 10 was used for SNP clustering for each LG. Finally, 2 or 3 SNPs per one Mbp were selected for mapping in order to obtain a genetic distance between markers <2 cM. Subsequently, only the first or second round of regression mapping was considered. Some SNPs with an unbalanced locus genotype frequency according to chi square distribution were eliminated, and each LG was recalculated. The coding of each SNP corresponds to a position in the Peach genome v.1.0 (Verde et al., 2013).

### Fruit quality trait association and QTL identification

Marker-trait association analyses between SNPs and agronomic traits were performed using TASSEL v5. A General Lineal Model (GLM) using numeric data joined to genotype data and principal component analysis (PCA) was used for generating Manhattan plots for each trait and year. In addition, a Mixed Lineal Model (MLM) was applied using kinship data to define the relationship between individuals, because MLM sometimes has higher statistical power than GLM and may detect more true associations. The width of the QTL intervals was calculated by interval-mapping (a parametric test) and the Kruskal–Wallis test (a non-parametric test) using MAPQTL v4. LOD significance thresholds were determined for each trait and year with a test of 1,000 permutations between phenotypic and genotypic data using the “Permutation Test” function. The strongest marker cofactors of each QTL were identified by “Automatic Cofactor Selection” using SNP markers that exceeded the LOD significance threshold as the most significant marker. Linkage maps and QTL intervals were drawn using MapChart 2.3 software (Voorrips, 2002).

## Results

### Transmission and correlations of fruit quality traits in Japanese plum

**Figure 1:**
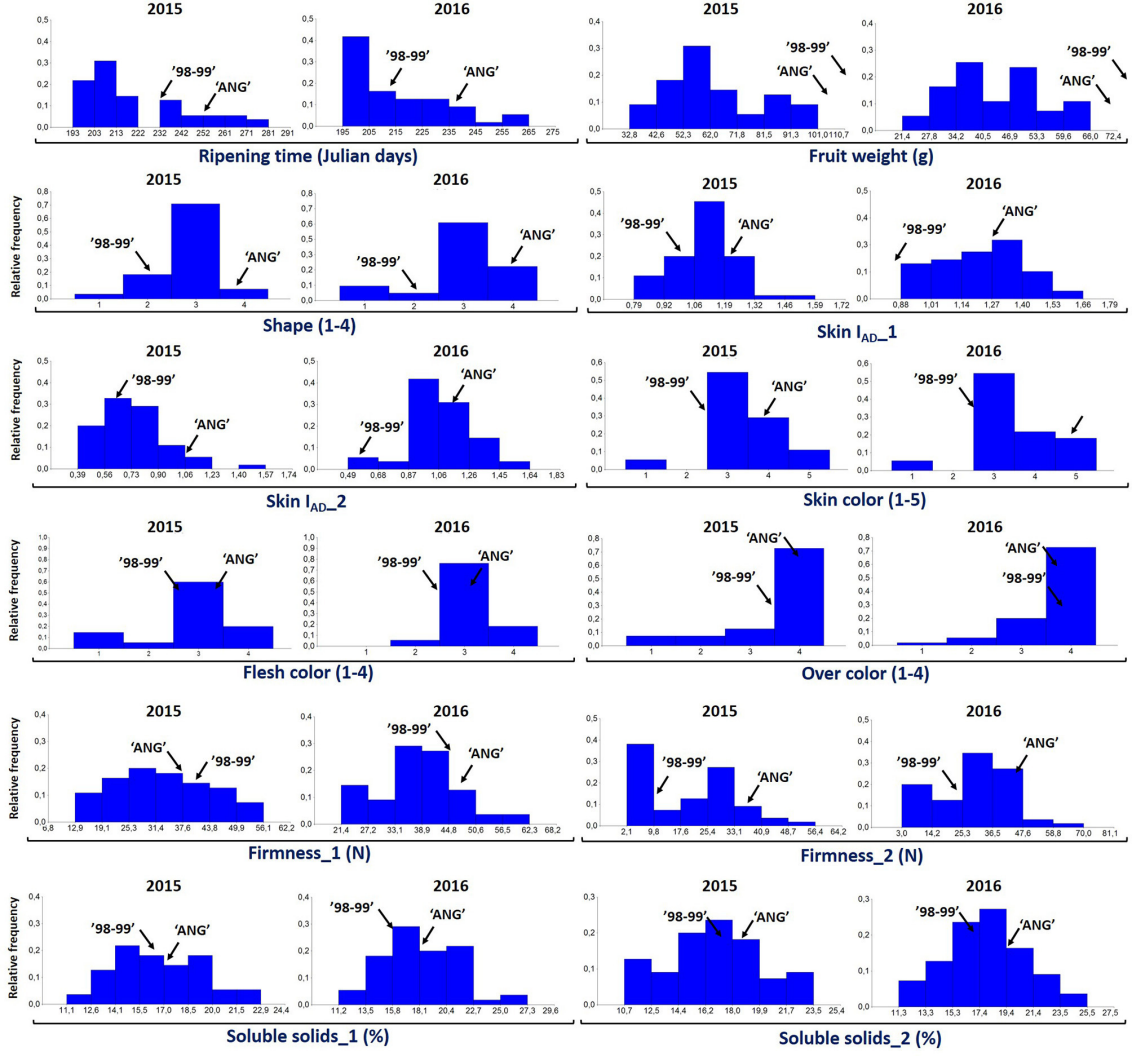
**Distribution of proportion of the 55 seedlings of “98–99” × “Angeleno” F1 Japanese plum progeny evaluated for different fruit quality traits including ripening time (Julian days), fruit weight (g), fruit shape (elongated, 1; hearted, 2; spherical, 3; oblate, 4), chlorophyll index (I_**AD**_), flesh color (white, 1; green, 2; yellow, 3; red, 4), skin color (green, 1; yellow, 2; red, 3; purple, 4; black, 5), firmness (N), over color (25%, 1; 50%, 2; 75%, 3; 100%, 4) and soluble solids content (%) in the years 2015 and 2016**.

For all the evaluated fruit quality traits we observed a great variability and noticeable segregation in the progenies (Figure 1). FW, IAD, FIRM, and SSC histograms revealed normal distribution according to Shapiro-Wilks test, indicating a polygenic nature and quantitative inheritance of these traits (Table S1), as previously reported in other *Prunus* species (Ruiz et al., 2010). RT histograms tended toward bimodal distribution especially for 2015 with mid-early and late-harvest seedlings, similar to findings in other *Prunus* species like peach (Eduardo et al., 2011; Pirona et al., 2013). SHP, SKC, FLSC, and OVC showed more categorical distributions (or Mendelian), due to the used phenotyping scale. FW ranged from 30 to 100 g in 2015 and between 20 and 70 g in 2016, while SSC_1 ranged from 10 to 23% for both years, and there were no apparent differences with SSC_2 values (Figure 1 and Table S1). For SHP most seedlings showed a spherical shape. Regarding fruit color, most frequent categories were red (3) and purple (4) skin color and yellow flesh, like their parents. Regarding OVC, most seedlings showed full color coverage (Figure 1). In the ANOVA for FW, I_AD_, FIRM, and SSC, there were significant differences for the genotype and year factors, suggesting a significant environmental influence on these traits, as largely reported in *Prunus* (Eduardo et al., 2011). In the Kruskal-Wallis analysis, by the other hand, categorical traits such as RT, SKC, FLSC, and OVC did not show significant differences (*p* > 0.01) between years, suggesting that these traits might be mostly influenced by genotype, rather than the year effect (Tables 1, 2).

**Table 1:** **ANOVA analysis for the years 2015 and 2016 for different fruit quality traits of Japanese plum “98–99” × “Angeleno” progeny (***n*** = 55), including ripening time, fruit weight, chlorophyll index (I_**AD**_), firmness, and soluble solids content**.

**Factors**	**Traits**	**Sum of squares**	**Df**	**Mean square**	***F***	**Sig**.
Genotype	Fruit weight	92326.36	54	1709.75	23.26	<0.0001
	I_AD__1	13.32	54	0.25	9.04	<0.0001
	Firmness_1	34463.77	54	638.22	10.71	<0.0001
	Soluble solids_1	4614.72	54	85.46	17.43	<0.0001
	I_AD__2	20.30	54	0.38	13.18	<0.0001
	Firmness_2	85634.74	54	1585.83	17.48	<0.0001
	Soluble solids_2	4446.48	54	82.34	17.94	<0.0001
Year	Fruit weight	46942.76	1	46942.76	638.58	<0.0001
	I_AD__1	1.04	1	1.04	37.95	<0.0001
	Firmness_1	2064.63	1	2064.63	34.66	<0.0001
	Soluble solids_1	238.75	1	238.75	48.70	<0.0001
	I_AD__2	12.25	1	12.25	429.72	<0.0001
	Firmness_2	12871.32	1	12871.32	141.89	<0.0001
	Soluble solids_2	98.08	1	98.08	21.37	<0.0001

*Fruit weight was evaluated at the harvest date, while I_AD_, firmness and soluble solids were evaluated at two maturity states, at the harvest date (_1) and 1 week after harvest (_2)*.

**Table 2:** **Kruskal-Wallis analysis for the years 2015 and 2016 for different agronomic traits of Japanese plum “98–99” × “Angeleno” progeny (***n*** = 55), including ripening time, fruit shape, skin color, flesh color, and over color evaluated at the harvest date**.

**Factor**	**Trait**	**Average**	***SD***	**Median**	**H**	***p***
Genotype	Ripening time	218.44	8.38	212	95.43	0.0004
	Shape	2.93	0.23	3	392.92	<0.0001
	Skin color	3.43	0.16	3	472.45	<0.0001
	Flesh color	3.11	0.14	3	282.36	<0.0001
	Over color	3.45	0.41	4	282.21	<0.0001
Year	Ripening time	218.44	20.41	212	0.28	0.5923
	Shape	2.915	0.73	3	10.59	0.0002
	Skin color	3.405	0.91	3	0.12	0.6976
	Flesh color	3.095	0.55	3	0.05	0.7632
	Over color	3.44	0.90	4	0.21	0.5919

Regarding the correlations between years, the highest values were found for RT, FW, and SKC (0.74, 0.64, and 0.80, respectively), all of which were significant with *p* < 0.005 (Table 3). Other phenotypic studies in peach (Quilot et al., 2004; Eduardo et al., 2011) and apricot (Ruiz et al., 2010; Salazar et al., 2013) show especially high inter-annual correlations for RT and SKC, but not so much for FW. These high correlations between years reveal that the genetic effect from genotypes is greater than the environmental effect for these traits, which makes them well-suited to being improved. In addition, the genetic value of each genotype was calculated using BLUP coefficients for the main correlated traits between years. Negative coefficients were found for early ripening seedlings (mid-January), smaller size (30 g), green and red skin color and lower SSC (12%), while positive coefficients were found for later ripening seedlings, bigger size (80 g), purple and black skin color and higher SSC (24%), as expected (Figure S1).

**Table 3:** **Pearson correlation coefficients for different agronomic traits for the years 2015 (below diagonal) and 2016 (above diagonal) in 55 “98–99” × “Angeleno” plum seedlings**.

	**RT**	**FW**	**SHP**	**I_AD__1**	**SKC**	**FLSC**	**OVC**	**FIRM_1**	**SSC_1**	**I_AD__2**	**FIRM_2**	**SSC_2**
RT	0.74^***^	0.44^***^	−0.21	−0.01	−0.52^***^	−0.27^*^	−0.75^***^	0.58	−0.13	0.13	0.60^***^	−0.36^**^
FW	0.35^**^	0.64^***^	−0.46^***^	−0.03	−0.23	−0.03	−0.32^*^	0.08	−0.46^***^	−0.14	0.12	−0.45^***^
SHP	−0.06	−0.05	0.38^***^	−0.06	0.25	−0.04	0.10	0.21	0.32^*^	−0.02	0.17	0.33^**^
I_AD__1	0.39^***^	−0.02	0.11	**0.20**	0.13	−0.02	−0.06	−0.07	0.13	0.67^***^	−0.09	0.07
SKC	−0.21	0.27^*^	0.03	−0.17	0.80^***^	0.11	0.57^***^	−0.28^*^	0.42^***^	0.06	−0.28^*^	0.51^***^
FLSC	−0.21	0.06	0.19	−0.14	0.31^*^	**0.22**	0.25	−0.22	−0.03	0.04	−0.23	0.04
OVC	0.01	0.20	−0.09	−0.18	0.37^***^	0.06	0.37^**^	−0.40^***^	0.02	−0.10	−0.36^**^	0.18
FIRM_1	−0.03	−0.36^**^	0.18	−0.01	−0.44^***^	−0.19	−0.26	0.33^*^	−0.01	0.14	0.84^***^	−0.18
SSC1	0.33^**^	0.08	0.04	0.17^*^	0.33^*^	0.14	0.05	−0.13	**0.35**	0.16	−0.08	0.93^***^
I_AD__2	0.38^**^	0.14	0.02	0.32	0.17	0.10	0.34^**^	−0.19	0.17	0.59^***^	0.31^*^	0.03
FIRM_2	0.21	−0.27^*^	0.00	−0.04	−0.54^***^	−0.11	−0.13	0.53^***^	−0.07	0.16	0.30^*^	−0.24
SSC_2	0.37^**^	0.08	−0.12	0.22	0.28^*^	0.11	0.07	−0.12	0.75^***^	0.22	0.01	0.35^**^

*RT, Ripening time; FW, fruit weight; SHP, shape; I_AD_, chlorophyll index; SKC, skin color; FLSC, flesh color; OVC, over color; FIRM, firmness; SSC, soluble solids content. All the traits were evaluated at the harvest date, while I_AD_, firmness and soluble solids were evaluated at two maturity states, at the harvest date (_1) and 1 week after harvest (_2)*.

* *P < 0.05*,

** *P < 0.01*,

*** *P < 0.005*.

### Genotype by sequencing (GBS)

Three FASTQ files (1 per plate) were generated, and over 220 million reads were read per lane, resulting in over 16 million tags for each plate or lane. This made it possible to get 2,272,102 good barcoded reads per each individual (Table S2). Similar results have been obtained in other *Prunus* species, with an average number of reads per genotype of 1,843,261 in cherry (Guajardo et al., 2015) and 2,333,869 in peach (Bielenberg et al., 2015). Finally, 5,230,374 tags were obtained after merging, and only three samples were failures (1.04%). Of these tags, 2,244,856 (42.9%) were aligned to unique positions, 190,411 (3.6%) were aligned to multiple positions and 2,795,107 (53.4%) could not be aligned in the peach genome v1 (Table S2). According to the HapMap files (SNP calls), 102,992 unfiltered SNPs and 42,909 filtered SNPs were counted, while in the VCF files, 744,927 SNPs were obtained (Table S3). In these VCF files, individual and site mean depth value reached 15 and 14, respectively, while both the individual and site missingness were around 0.14 (Table S4).

### Linkage analysis and identification of QTLs linked to fruit quality traits in Japanese plum

A total of 981 SNPs were mapped in the “98–99” × “Angeleno” progeny, with 479 SNPs for “98–99” and 502 SNPs for “Angeleno” covering a genomic region of 688.8 and 647.03 cM, respectively (**Figure 3**). This implies a mean genetic distance between markers of below 2 cM. As expected, the LG1 was the longest chromosome with 156.3 cM in “98–99” and 153.1 cM in “Angeleno.” LG5 was the shortest chromosome, with 61.5 cM in “98–99” and 79.8 cM in “Angeleno.” This supposes close to 45 Mbp for LG1 and below 20 Mbp for LG5 (Verde et al., 2013). The type of segregation was of 232 SNPs (23.7%) for the female parent (<lmxll>), 324 SNPs (33%) for the male parent (<nnxnp>) and 425 heterozygous SNPs (43.3%) for both parents (<hkxhk>), agreeing with the higher heterozygosity observed in “Angeleno” respect to “98–99.” These values were similar in some cherry progenies using GBS (Guajardo et al., 2015). GBS has also been used in F2 peach progenies (Bielenberg et al., 2015), and the map distance obtained was 666.1 cM, similar to that reached in this study. The genetic marker distance, however, was around 2.85 cM, which was bigger than that observed in the “98–99” × “Angeleno” genetic map (1.48 and 1.28 cM).

**Figure 2:**
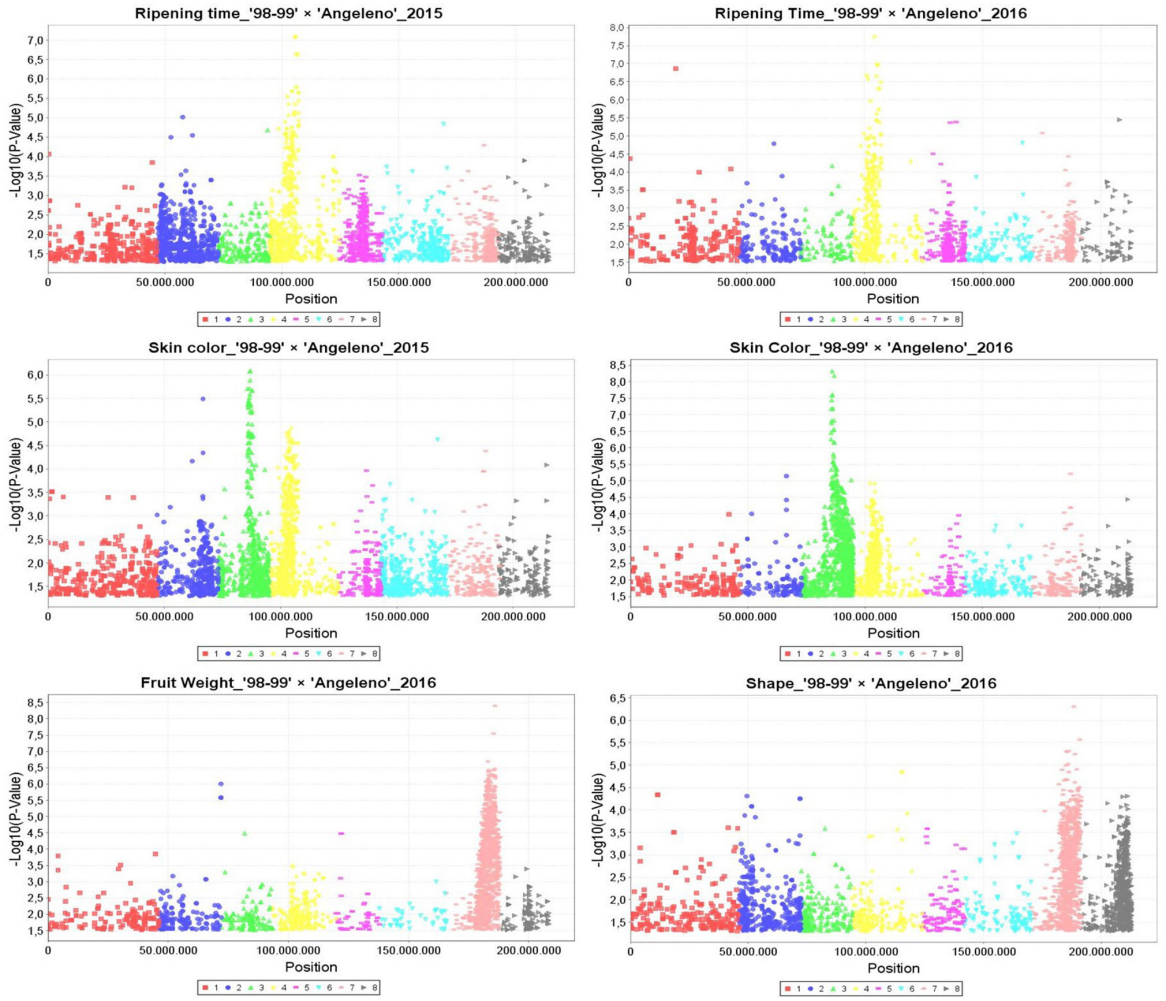
**Manhattan plots calculated by General Linear Model (GLM) in TASSEL5 for the main QTLs identified linked to ripening time, skin color, fruit weight, and fruit shape in the years 2015 and 2016**. Vertical axis are indicating the −Log_10_ (*p*-value) and horizontal axis are indicating the position in millions pair of bases of each SNP aligned to Peach genome v1.

Manhattan plots were generated according to marker-trait association by GLM for the years 2015 and 2016, identifying hundreds of SNPs related with RT in LG4, with SKC in LG3 and LG4 and with FW and SHP mainly in LG7 (Figure 2). These results were confirmed by MLM, which detected the main QTLs for RT in LG4 at around 8 and 11 Mbps and those for SKC in LG3 between 12 and 15 Mbps for both years. The FW QTL, on the other hand, was located in LG7 at around 16 to 20 Mbps in 2016. All of these traits, especially RT and SKC, reached a phenotypic explanation variance (PEV) of around 30–40% (Tables S7–S9).

**Figure 3:**
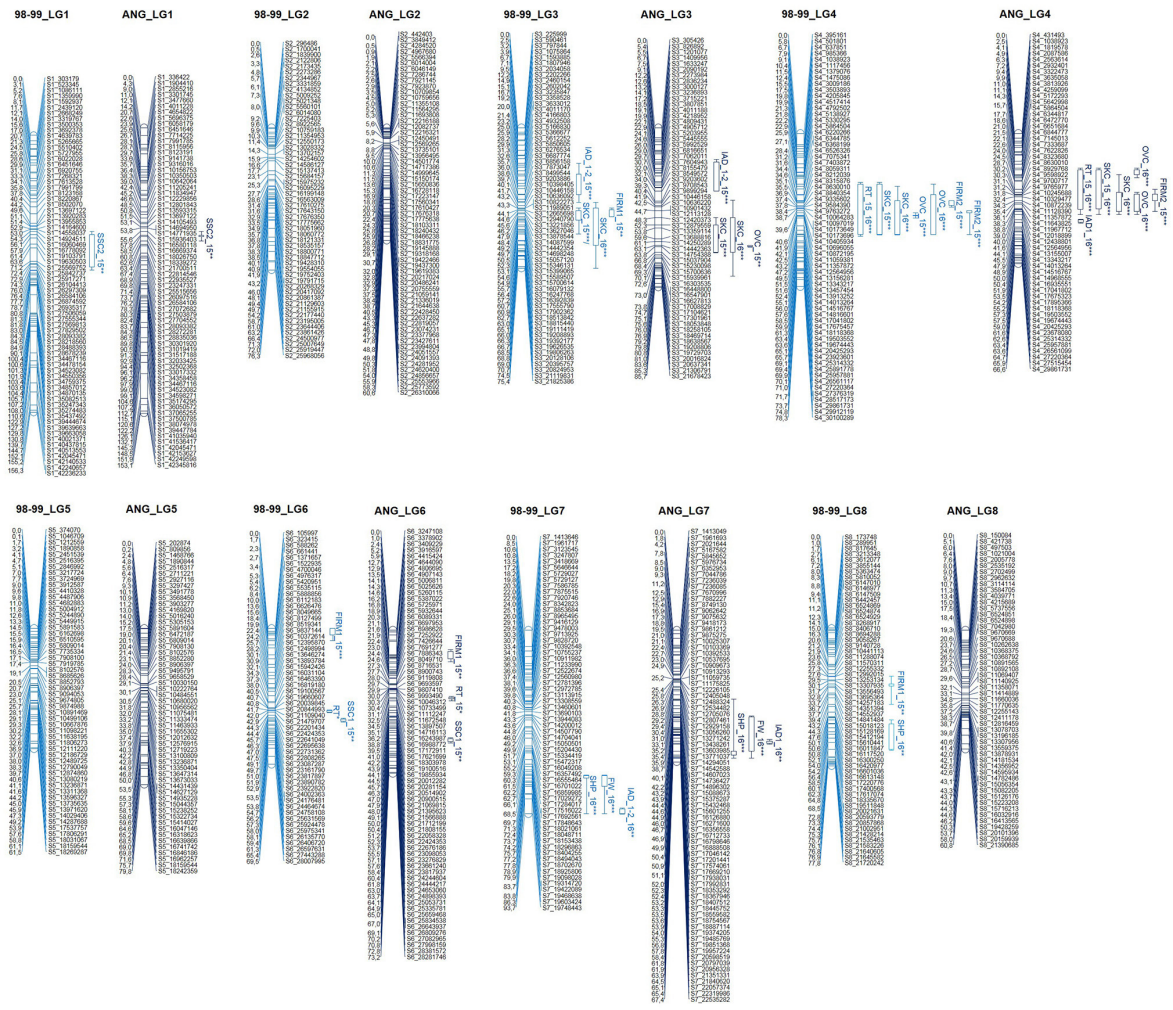
**Genetic maps of Japanese plum “98–99” (female) and “Angeleno” (male) and QTL identification by interval mapping analysis for two years of phenotyping: 2015 (light blue) and 2016 (dark blue)**. RT, Ripening time; FW, fruit weight; SHP, shape; I_AD_, chlorophyll index; SKC, skin color; FLSC, flesh color; OVC, over color; FIRM, firmness; SSC, soluble solids content. LOD threshold for QTL intervals: ^*^α < 0.10, ^**^α < 0.05, and ^***^α < 0.01.

As for the QTL mapping analysis, several QTLs (including those previously mentioned) were identified by IM and KW for 2015 and 2016. The majority of these QTLs were localized in LG3, including I_AD__1-2, SKC, and OVC, and in LG4, including RT, SKC, OVC, and FIRM. SSC QTLs were identified in LG1 and LG6 (Table 4 and Figure 3). The most important QTLs identified were for RT, SKC and FW. According to IM, the RT QTL was strongly localized in LG4 between 6 and 12 Mbp with a LOD value over 5 (LODα0.01; Figure 3). This long QTL interval is due to the fact that there are many SNPs co-localizing in LG4, indicating that this chromosome has a strong influence on this trait, as mentioned above (Figure 2). In addition, this QTL reached a maximum LOD value of 8.45 and a PEV value of over 40% for both years (Table 4 and Tables S5, S6). S4_11357872 and S4_12564956 were selected as cofactors for “98–99” in 2015 and 2016, while a set of five cofactors from S4_9765977 to S4_11967712 were selected for “Angeleno” in 2016, with S4_11967712 reaching a PEV value of 84.9% (Table S6). The heterozygous genotypes (np in SNP S4_11967712) from “Angeleno” present on average earlier ripening time and the homozygous (nn) with later ripening time, with an average difference of close to 30 days in 2015 and 20 days in 2016.

**Table 4:** **QTL analysis by interval mapping (parametric test) and mapping information for fruit quality traits in a F1 Japanese plum progeny of “98–99” × “Angeleno.”**.

**Trait**	**Year**	**Parent**	**QTL**	**LG**	**IM_95% (cM)**	**Location**	**Nearest marker**	**LOD**	**PEV**
Ripening time	2015	98–99	RT	4	[25.0; 52.4]	43.2	S4_11357872	6.73	46.3
	2015	ANG	RT	4	[17.7; 41.6]	36.1	S4_11357872	7.53	51.6
	2016	98–99	RT	4	[25.0; 52.0]	43.2	S4_11357872	6.51	63.2
	2016	ANG	RT	4	[17.0; 41.6]	36.1	S4_11357872	8.45	63.5
Chlorophyll index	2015	98–99	I_AD__1-2	3	[19.2; 40.1]	35.7	S3_6856158	4.82	34.8
	2015	ANG	I_AD__1-2	3	[17.2; 34]	32.1	S3_8549572	5.14	39.4
Skin color	2015	98–99	SKC	3	[43.2; 57.2]	49.7	S3_14698248	6.61	43.3
	2015	98–99	SKC	4	[25.0; 52.0]	42.1	S4_10872195	6.54	44.8
	2015	ANG	SKC	3	[48.3; 67.0]	53.8	S3_13359114	7.07	44.7
	2015	ANG	SKC	4	[17.7; 41.6]	31.1	S4_9700717	5.16	35.9
	2016	98–99	SKC	3	[40.1; 75.4]	45.6	S3_13221856	5.12	34.9
	2016	98–99	SKC	4	[27.1; 52.0]	42.1	S4_10872195	5.56	41.3
	2016	ANG	SKC	3	[39.0; 79.8]	52.3	S3_12879559	8.15	50.4
	2016	ANG	SKC	4	[21.0; 46.0]	38.8	S4_11967712	6.04	40.1
Over color	2015	98–99	OVC	4	[41.9; 42.1]	41.9	S4_10696055	5.1	33.3
	2015	ANG	OVC	3	[63.1; 63.8]	63.8	S3_14754388	3.68	67.6
	2016	98–99	OVC	4	[25.9; 59.6]	39.6	S4_10173649	9.21	55.6
	2016	ANG	OVC	4	[17.7; 41.6]	38.8	S4_11967712	8.01	48.9
Soluble solids	2015	98–99	SSC_2	1	[56.1; 74.3]	63.6	S1_19630503	3.70	27.1
	2015	98–99	SSC_1	6	[46.3; 47.5]	47.5	S6_22808265	3.61	31.3
	2015	ANG	SSC_2	1	[57.8; 58.5]	58.5	S1_16669374	3.71	27.8
	2015	ANG	SSC_1	6	[55.5; 57.0]	57.0	S6_23276829	4.25	28.7
Fruit weight	2016	98–99	FW	7	[74.0; 93.7]	74.2	S7_18296863	5.76	47.9
	2016	ANG	FW	7	[44.0; 65.4]	58.4	S7_20598519	5.79	39.0
Shape	2016	98–99	SHP	7	[74.0; 93.7]	74.2	S7_18296863	4.75	42.5
	2016	ANG	SHP	7	[35.2; 65.4]	61.9	S7_20956328	4.73	36.1

*LG, linkage group; LOD, logarithm of odds; IM, interval mapping at LOD threshold ≤ 0.05*.

Regarding the SKC QTL, LG3, and LG4 were the LGs most linked to this trait for both parents and years (Tables 4 and Tables S5, S6). The SKC QTL co-localized in LG4 in the same genomic region as RT for both years and in the same genomic region as the OVC QTL in 2016. This may be due to the pleiotropic effect of certain genes that are simultaneously related to maturity date and fruit skin color, as has been described by other authors like Eduardo et al. (2011). For both QTLs, the heterozygous allele from “Angeleno” tended toward purple SKC (4) and 100% OVC (4). The occurrence of the OVC QTL only in 2016 may be explained by an environmental effect. The increased insolation in that year caused the fruits to obtain a much fuller color, which we could also consider as a genetic effect, because the conditions in 2016 probably made it possible for the expression of this trait to be more intense. However, the most important QTL for SKC was strongly localized in LG3 between 52 and 61 cM, especially in “Angeleno” (Figure 3). Cofactors between 12 and 14 Mbp were selected in “Angeleno” for both years, and the most significant was S3_12879559 in 2016, which reached over 80% PEV. This means that the seedlings showing heterozygous genotypes on SNPs segregating for “Angeleno” have a darker color than homozygous genotypes, whose fruits tended to have a red skin color (phenotypic mean of 4.11 in np seedlings vs. a mean of 2.85 in nn seedlings; Table S6). In addition, alleles from “98–99” in this LG3 QTL, although with less clear allele effects among years, show also to contribute significantly to the trait variability in this progeny.

In addition, a QTL was identified in LG3 related to the chlorophyll index calculated as the difference between I_AD_ at different maturity states (I_AD__1-2). For this QTL, S3_4166803 and S3_8549572 were identified as the cofactors of “98–99” and “Angeleno,” respectively. The explained phenotypic variance was over 30% for both parents (Tables 4 and Tables S5, S6). These QTLs values are undoubtedly due to the significant difference between both parents in terms of chlorophyll degradation. The cultivar “98–99” undergoes greater chlorophyll degradation than “Angeleno,” a suppressed climacteric cultivar (Singh et al., 2012), which could be related to the higher softening rate of “98–99.”

Moreover, QTLs for FW and SHP were also identified in 2016, which co-localized in the same position in the LG7 of “Angeleno,” between 16 and 20 Mbp (Figure 3 and Table 4). These QTLs may be related to the correlation between FW and SHP in the same year (Table 3), which indicates that lower fruit weight is related to more elongated fruits. Nevertheless, we must emphasize that this correlation is not very high because there is no clear segregation between spherical (3) and elongated fruits (1). Both QTLs are thus related to fruit size, and in both cases the PEV value is close to 40%. This means an average fruit weight of above 50 g or below 40 g depending on the allelic class, with S7_20598519 being the most important cofactor (Tables S5, S6). Contrary to ripening time, QTLs for fruit size and weight do not appear to be strongly linked to a particular linkage group.

Finally, as for SSC, two QTLs were identified in LG1 and LG6 for 2015 (Figure 3 and Table 4). The SSC QTL at maturity completion (SSC_2) for “98–99” was localized at around 54–76 cM, and S1_19630503 was selected as a cofactor, reaching a PEV value of around 25% of difference between phenotypic classes (15.5–18.5%, Table S5). To the contrary, the second QTL for SSC at harvest (SSC_1) for “Angeleno” was identified in LG6 at around 55–57 cM and S6_23276829 was selected as a cofactor, reaching a PEV value of close to 30%. In this case, S6_23276829 determines three phenotypic classes from ranging 14.5 to 18.4 (Table S6).

## Discussion

Assisted selection using molecular markers is not an easy path for improving the quantitative traits in any plant species, so it is often preferred to study qualitative traits, which have more easily predictable behavior and are generally associated with few genes. Improving fruit quality, however, is one of the priority aims for any breeding program, which is a great challenge because the majority of quality traits are quantitatively inherited. Ripening time, fruit size, soluble solids content, acidity and antioxidant and functional-food compounds related to fruit color are perhaps the most desirable traits for improving fruits from the point of view of phenology, organoleptic quality and health. However, post-harvest fruit behavior is also highly important, and increasing our knowledge of all the factors involved in the fruit ripening process may provide us with molecular tools for selecting seedlings with less perishable fruits and therefore a longer useful life.

The use of NGS-based molecular tools such as GBS demonstrate the great importance of genomes sequencing, which has been successfully implemented in our F1 plum population, generating very saturated parental genetic maps with a mean distance between markers <2 cM. Therefore, GBS is profiled as an appropriate application tool for breeding programs of this species, allowing a greater genetic depth and more accurate QTL detection. Moreover, the results obtained in the “98–99” × “Angeleno” progeny showed similar behavior to other *Prunus* species and provide promising information about QTLs from a phenological point of view, especially regarding ripening time and fruit quality traits like fruit weight, fruit skin color and soluble solids content. Among these mentioned traits, in our study we determined the occurrence of QTL of strong and consistent effect through harvest seasons for ripening time, skin color, and chlorophyll degradation.

Ripening time QTL is undoubtedly important as it can help us select early and late seedlings according to our needs. In the studied F1 progeny, MD showed a bimodal distribution, indicating that segregation of few loci of major effect and an undetermined number of loci of minor effect for this trait. In most of reported segregations of this trait in other F1 progenies of *Prunus* species, a continuous segregation was observed (e.g., “BxO” in Eduardo et al., 2011, and “CxEL” in Pacheco et al., 2014), supposedly depending in the genetic backgrounds of the parents. Most seedlings (around a 50%) show mid-early ripening dates of 215 or less Julian days, suggesting a dominance effect from alleles of “98–99” parent. Moreover, the occurrence of this QTL is highly conserved within *Prunus* species, being related the MD locus with NAC family transcription factors (Pirona et al., 2013) which are involved in abiotic stress tolerance (Shen et al., 2017). In our population cofactors between S4_9765977 to S4_11967712 were identified in the same genomic region of MD locus. The location of this QTL has been previously reported in the most important commercial *Prunus* species such as peach, apricot, and cherry (Eduardo et al., 2011; Dirlewanger et al., 2012; Pirona et al., 2013; Salazar et al., 2013, 2016; Nuñez-Lillo et al., 2015). We can therefore assert that this QTL occurs in a genomic region that is highly conserved in different *Prunus* species. Further studies on this genomic region in F2 peach populations have allowed the location a *MD* locus of 220 kb (ppa008301m) as a candidate gene encoding transcription factors of the *NAC* family (Pirona et al., 2013). Another more recent study in peach and nectarine localized the same QTL in LG4 at around 31 to 42 cM and identified five candidate genes related to maturation date and nine candidate genes for flesh mealiness, suggesting *ANAC072* and ppa010982m (*ERF4*) as transcription factors (TFs) involved in the expression of both traits (Nuñez-Lillo et al., 2015). Nuñez-Lillo et al. (2015) and Eduardo et al. (2015) suggest that mealiness and slow ripening could be related with maturation date, which leads us to study these traits together in following phenotyping seasons, in order to decipher the biological processes underlying this strong pleiotropic effect. Eduardo et al. (2015) reported that the slow ripening trait is determined for a single gene (Sr/sr) and that this gene is mapped in the same region of the MD locus in peach. The MD locus is therefore partly responsible for maturity date variability and could be related with the sr allele, which could confer desirable postharvest traits such as slow ripening.

Regarding SKC trait in the studied cross, most seedlings had fruits with red or violet colors, suggesting a dominant effect of alleles from darker parent (“Angeleno”). This strong effect was confirmed by the finding QTLs segregating in both parents, on two harvest seasons and located in a region that spans between positions 10,446,158 and 14,698,248 of chromosome 3. Marker showing the most significant effect in trait variation was S3_13359114, segregating only in “Angeleno” (Expl% = 41.9–85.5). Other important component of this trait corresponded to the QTLs mostly detected in “98–99” parent, and co-localizing with ripening time QTLs. Several QTLs for fruit skin color have been reported for peach in different LGs: in LG2 (Quarta et al., 2000); in LG2 and LG6 (Verde et al., 2002); in LG5 (Quilot et al., 2004); and in LGs 3, 4, 6, and 7 (Eduardo et al., 2011). Moreover, Dirlewanger et al. (2004b) and Martínez-García et al. (2013b) identified QTLs for red and yellow flesh color in LG1. However, as occurred for RT in LG4, the SKC QTL in LG3 seems to have a greater effect given that other authors have identified major QTLs in this LG in different *Prunus* species, indicating that the occurrence of this QTL may be highly conserved within *Prunus* species (Sooriyapathirana et al., 2010; Socquet-Juglard et al., 2013; Frett et al., 2014). In cherry, for example, Sooriyapathirana et al. (2010) localized a major QTL for skin and flesh color in LG3 at around 54 cM with a PEV value of around 80%. This QTL co-localized in the same interval in the “98–99” × “Angeleno” progeny. In addition, *PavMYB10*, a homologous gene to apple and Arabidopsis, was identified as a candidate gene (Allan et al., 2008). In apricot, Socquet-Juglard et al. (2013) also identified an important QTL for ground color in LG3, and the nearest marker was located at around 55 cM. Frett et al. (2014) also localized a similar QTL for blush in LG3, identifying candidate genes involved in the flesh coloration of peach (*PprMYB10*), cherry (*PavMYB10*) and apple (*MdMYB1*/*MdMYBA*/*MdMYB10*). In apple, several authors claim that anthocyanin biosynthesis is regulated by MYB transcription factors that are positively correlated with red skin color (Takos et al., 2006; Ban et al., 2007; Chagné et al., 2007; Allan et al., 2008). Moreover, the SNP S3_12879559 is co-localizing close to a MYB transcription factor in our population (Table S10). This suggests similar genetic control of fruit skin color within rosaceous species. In *P. salicina* (González et al., 2016) several EST-SSRs have recently been developed from genes coding for proteins involved in the flavonoid pathway, in order to explore the genetic structure of 29 accessions. According to peach genome v1 (Verde et al., 2013), most of these SSRs are located between positions 12,842,509 and 12,842,693 of chromosome 3, close to the nearest marker for the SKC QTL from the “98–99” × “Angeleno” population, suggesting that the same genes could be involved in fruit skin color and the flavonoid pathway, and confirming that flavonoid pathway comprises molecular processes that are of key importance in the genetic control of skin color trait in rosaceous species. Importantly, the chlorophyll degradation QTL identified upstream to skin color QTL of LG3 offers to the community a new approach for the identification of seedlings potentially with a good postharvest behavior because a faster chlorophyll degradation is often related to more abrupt fruit senescence and fruit softening. As reported by Wu et al. (2011), chlorophyll degradation is related to decrease in polygalacturonase and pectin methylesterase, given that chlorophyll variation is an indicator of fruit ripening and senescence. Further studies are required for fine mapping of this region and identification of genes involved in this physiological process of great commercial importance.

FW QTL located in the LG7 means a promising QTL because usually fruit weight or fruit size are strongly influenced by climatic conditions, with a very polygenic nature, as reported in *Prunus* species for different LGs. In fact, contrary to ripening time, QTLs for fruit size and weight do not appear to be strongly linked to a particular linkage group. For example, in peach, Quilot et al. (2004) placed different fruit diameter QTLs in LGs 1, 4, 5, 6, and 7 and fruit weight QTLs in LGs 1, 2, 4, 5, and 7, while Cantín et al. (2010) located the fruit diameter QTLs in LG4. Also in peach, Eduardo et al. (2011) located a fruit weight QTL in LG6, while in cherry, Zhang et al. (2010) identified fruit size QTLs in LG2 and LG6. More recently, Campoy et al. (2015) located major QTLs linked to sweet cherry fruit size control in LG5 and identified different candidate genes along the QTL interval. Earlier, De Franceschi et al. (2013) identified 23 genes from Cell Number Regulator (CNR) progeny related to FW in cherry in LG2 and LG6, and they found PavCNR12 and PavCNR20 in the QTL intervals. Genes from CNR progeny may thus control fruit size by increasing the cell number and organ size in *Prunus* species as well as in tomato and maize (Frary et al., 2000; Guo et al., 2010). In a F2 peach population, Da Silva Linge et al. (2015) recently identified several QTLs related to FW in different LGs. The most important QTL was localized in LG7, and it showed a LOD value over 10 and a PEV value of around 20%. The nearest FW QTL marker was located at around 40 cM, similar to findings in the “98–99” × “Angeleno” progeny. To the contrary, in an F1 nectarine population, Zeballos et al. (2016) located the most important QTLs for FW in LG4, with a maximum PEV value of around 50%. In cherry, Rosyara et al. (2013) identified six QTLs for fruit size in LG1, LG3, and LG6 with the Bayesian approach, using different progenies and their ancestors simultaneously. Taking a similar approach in peach, Fresnedo-Ramírez et al. (2016) were also able to identify several QTLs for fruit diameter and fruit weight along the genome, supporting the fact that, although its complexity, actually available genomic, genetic and statistical tools allow the genetic dissection of this trait. Finally, several authors have described QTLs related to SSC and sugar metabolism (fructose, glucose, and sucrose) along the *Prunus* genome, especially in intraspecific and inter-specific peach crosses (Dirlewanger et al., 1999; Quarta et al., 2000; Etienne et al., 2002; Verde et al., 2002; Quilot et al., 2004; Eduardo et al., 2011; Illa et al., 2011). This indicates the polygenic character of this trait linked to different chromosomes. Recently, Desnoues et al. (2016) studied QTLs related to sugar metabolism during fruit development in peach. These authors identified five QTLs for sucrose and located the most important QTL in LG1, with a percentage of trait variability explained of between 40 and 80%. These authors also identified the following four candidate genes: *SUGAR TRANSPORTER* (Prupe.1G133300); *INVERTASE INHIBITOR* (Prupe.1G131900, Prupe.1G132000, Prupe.1G132300); and *SUSY* (Prupe.1G131700).

## Conclusion

In this work we reported genomic regions strongly correlated with important fruit quality traits such as ripening time (RT), chlorophyll degradation index (I_AD__1-2), skin color (SKC), over color (OVC), soluble solids content (SSC), weight (FW), and shape (SHP). QTLs with a significant and consistent effect through years were found for RT, I_AD__1-2, and SKC. Due to the complexity of these polygenic traits, it will be necessary to carry out a more detailed study in order to validate the reported QTLs, considering the phenotyping of more seedlings, in order to include more recombinants, and thus obtain QTLs covering narrower genomic regions for fine mapping of the traits; moreover, more phenotyping years must be included to have a more precise estimation of genetic and seasonal effects on these traits. Moreover, new molecular markers specifically designed for each QTL region must be included in order to assist fine mapping of genes controlling these traits. Such further studies would make it possible to use MAS as a breeding tool for Japanese plum. Finally, from a postharvest point of view, non-destructive control of the firmness of the fruit during the critical maturation period (Infante et al., 2011b) would be necessary for investigating a possible relationship between the softening rate and a locus of the genome.

## Author contributions

JS: Writer and analyst of results. IP: Analyst of results and reviewer. PS: Management and technical support. PZ and CS: Laboratory technician. MA and DV: Adviser and rewiewer. DR and PM: Writer and reviewer. RI: Corresponding author and reviewer.

### Conflict of interest statement

The authors declare that the research was conducted in the absence of any commercial or financial relationships that could be construed as a potential conflict of interest.

## Supplementary material

Figure S1 **Best Linear Unbiased Predictors (BLUPs) coefficients of each seedling for ripening time, fruit weight, skin color, and soluble solids content**.

Table S1 **Summary of descriptive statistics and normality test of Shapiro-Wilks of the evaluated traits**.

Table S2 **Summary of FastQ files and alignment results obtained by GBS**.

Table S3 **Summary of SNPs obtained from calling SNPs**.

Table S4 **Depth and missingness from the unfiltered VCF file**.

Table S5 **Summary of the most important SNPs as cofactors related to different QTLs in the “98–99” parent for 2015 and 2016**.

Table S6 **Summary of the most important SNPs as cofactors related to different QTLs in the “Angeleno” parent for 2015 and 2016**.

Table S7 **Marker trait association by Mixed Linear Model (MLM) by TASSEL v5 in 2015**.

Table S8 **Marker trait association by Mixed Linear Model (MLM) by TASSEL v5 in 2016**.

Table S9 **Marker trait association by Mixed Linear Model (MLM) by TASSEL v5 for both years**.

Table S10 **Summary of nearest genes related to the most important QTLs identified (ripening time, RT; fruit weight, FW; and skin color, SKC)**.
