# Supplementary figures and images for: Genotyping by Sequencing for SNP-Based Linkage Analysis and Identification of QTLs Linked to Fruit Quality Traits in Japanese Plum (Prunus salicina Lindl.)
Source: Front Plant Sci. 2017 Apr 11;8:476. doi: 10.3389/fpls.2017.00476 (PMC5386982; doi:10.3389/fpls.2017.00476)

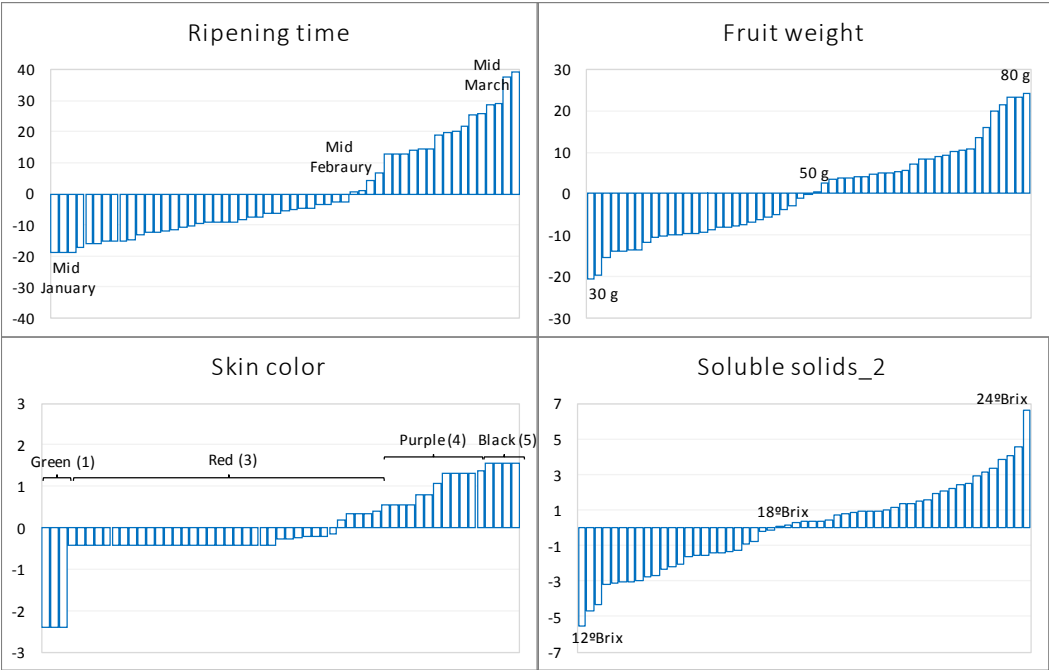

Supplement: Figure S1 — Best Linear Unbiased Predictors (BLUPs) coefficients of each seedling for ripening time, fruit weight, skin color, and soluble solids content. [file FigureS1.PDF]
