# Supplementary material for: Genotyping by Sequencing for SNP-Based Linkage Analysis and Identification of QTLs Linked to Fruit Quality Traits in Japanese Plum (Prunus salicina Lindl.)
Source: Front Plant Sci. 2017 Apr 11;8:476. doi: 10.3389/fpls.2017.00476 (PMC5386982; doi:10.3389/fpls.2017.00476)
Supplement: Table S1 — Summary of descriptive statistics and normality test of Shapiro-Wilks of the evaluated traits. [file Table1.DOCX]

**Table S1.** Summary of descriptive statistics and normality test of Shapiro-Wilks of the evaluated traits.

| Year | Trait | n | Average | SD | Min | Max | S-W | Sig.* |
| --- | --- | --- | --- | --- | --- | --- | --- | --- |
| 2015 | Ripening time | 55 | 220.73 | 23.89 | 193.00 | 281.00 | 0.86 | <0.0001 |
|  | Fruit weight | 157 | 63.53 | 18.76 | 30.02 | 120.39 | 0.93 | 0.0146 |
|  | Shape | 157 | 2.84 | 0.58 | 1.00 | 4.00 | 0.73 | <0.0001 |
|  | I_AD__1 | 157 | 1.12 | 0.16 | 0.78 | 1.71 | 0.98 | 0.8006 |
|  | I_AD__2 | 152 | 0.77 | 0.23 | 0.33 | 1.57 | 0.95 | 0.1121 |
|  | Skin color | 157 | 3.38 | 0.89 | 1.00 | 5.00 | 0.80 | <0.0001 |
|  | Flesh color | 157 | 3.07 | 0.64 | 1.00 | 4.00 | 0.73 | <0.0001 |
|  | Over color | 157 | 3.43 | 0.96 | 1.00 | 4.00 | 0.66 | <0.0001 |
|  | Firmness_1 | 145 | 34.39 | 10.80 | 12.93 | 63.49 | 0.96 | 0.3617 |
|  | Firmness_2 | 149 | 20.60 | 14.02 | 1.86 | 63.07 | 0.91 | 0.0020 |
|  | Soluble solids_1 | 157 | 16.67 | 3.25 | 8.60 | 24.60 | 0.96 | 0.2930 |
|  | Soluble solids_2 | 152 | 17.04 | 3.31 | 8.10 | 23.70 | 0.97 | 0.5010 |
| 2016 | Ripening time | 55 | 216.15 | 16.94 | 195.00 | 265.00 | 0.86 | <0.0001 |
|  | Fruit weight | 507 | 43.21 | 12.74 | 16.80 | 91.06 | 0.95 | 0.0773 |
|  | Shape | 498 | 2.99 | 0.89 | 1.00 | 4.00 | 0.75 | <0.0001 |
|  | I_AD__1 | 507 | 1.21 | 0.23 | 0.34 | 1.69 | 0.96 | 0.3274 |
|  | I_AD__2 | 495 | 1.09 | 0.25 | 0.27 | 1.82 | 0.98 | 0.9522 |
|  | Skin color | 498 | 3.43 | 0.94 | 1.00 | 5.00 | 0.80 | <0.0001 |
|  | Flesh color | 498 | 3.12 | 0.46 | 2.00 | 4.00 | 0.64 | <0.0001 |
|  | Over color | 507 | 3.45 | 0.84 | 1.00 | 4.00 | 0.73 | <0.0001 |
|  | Firmness_1 | 497 | 38.53 | 10.31 | 5.65 | 73.04 | 0.96 | 0.4195 |
|  | Firmness_2 | 490 | 30.49 | 15.08 | 0.94 | 78.80 | 0.95 | 0.2257 |
|  | Soluble solids_1 | 507 | 18.03 | 3.43 | 2.17 | 27.80 | 0.98 | 0.8264 |
|  | Soluble solids_2 | 495 | 17.91 | 3.34 | 10.20 | 28.70 | 0.98 | 0.8332 |

*Traits at P value>0.01 meet the normality criteria
