# Supplementary material for: Genotyping by Sequencing for SNP-Based Linkage Analysis and Identification of QTLs Linked to Fruit Quality Traits in Japanese Plum (Prunus salicina Lindl.)
Source: Front Plant Sci. 2017 Apr 11;8:476. doi: 10.3389/fpls.2017.00476 (PMC5386982; doi:10.3389/fpls.2017.00476)
Supplement: Table S2 — Summary of FastQ files and alignment results obtained by GBS. [file Table2.DOCX]

**Table S2.** Summary of FastQ files and alignment results obtained by GBS.

| FastQ file | Samples  per plate | Reads | Good barcoded reads |
| --- | --- | --- | --- |
| C615BACXX_1_fastq.gz | 96 | 258,570,867 | 195,821,190 |
| C615BACXX_2_fastq.gz | 96 | 248,878,182 | 229,358,555 |
| C615BACXX_3_fastq.gz | 91 | 227,881,369 | 217,825,123 |
| Total | 283 | 735,330,418 | 643,004,868 |
| Reads per individual |  | 2,598,340 | 2,272,102 |
| Alignment results |  |  |  |
| Tags aligned to unique positions | | | 2,244,856 (42.9%) |
| Tags aligned to multiple positions | | | 190,411 (3.6%) |
| Tags not aligned | | | 2,795,107 (53.4%) |
| Total tags | | | 5,230,374 |
