# Supplementary material for: Genotyping by Sequencing for SNP-Based Linkage Analysis and Identification of QTLs Linked to Fruit Quality Traits in Japanese Plum (Prunus salicina Lindl.)
Source: Front Plant Sci. 2017 Apr 11;8:476. doi: 10.3389/fpls.2017.00476 (PMC5386982; doi:10.3389/fpls.2017.00476)
Supplement: Table S3 — Summary of SNPs obtained from calling SNPs. [file Table3.DOCX]

**Table S3.** Summary of SNPs obtained from calling SNPs.

| Resulting SNPs | |
| --- | --- |
| VCF SNPs | 744,927 |
| HapMap SNPs (unfiltered) | 102,992 (100%) |
| HapMap SNPs (filtered) | 42,909 (41.7%) |
| SNPs LG1 | 8,433 (19.7%) |
| SNPs LG2 | 4,816 (11.2%) |
| SNPs LG3 | 4,939 (11.5%) |
| SNPs LG4 | 5,114 (11.9%) |
| SNPs LG5 | 4,254 (9.9%) |
| SNPs LG6 | 5,779 (13.5%) |
| SNPs LG7 | 4,935 (11.5%) |
| SNPs LG8 | 4,639 (10.8%) |
