# Supplementary material for: Genotyping by Sequencing for SNP-Based Linkage Analysis and Identification of QTLs Linked to Fruit Quality Traits in Japanese Plum (Prunus salicina Lindl.)
Source: Front Plant Sci. 2017 Apr 11;8:476. doi: 10.3389/fpls.2017.00476 (PMC5386982; doi:10.3389/fpls.2017.00476)
Supplement: Table S4 — Depth and missingness from the unfiltered VCF file. [file Table4.DOCX]

**Table S4.** Depth and missingness from the unfiltered VCF file.

|  | Mean | Median | SD |
| --- | --- | --- | --- |
| Individual depth | 15.001 | 14.804 | 5.684 |
| Site depth | 14.041 | 11.694 | 12.499 |
| Individual missingness | 0.140 | 0.113 | 0.128 |
| Site missingness | 0.140 | 0.031 | 0.247 |
