# Supplementary material for: Genotyping by Sequencing for SNP-Based Linkage Analysis and Identification of QTLs Linked to Fruit Quality Traits in Japanese Plum (Prunus salicina Lindl.)
Source: Front Plant Sci. 2017 Apr 11;8:476. doi: 10.3389/fpls.2017.00476 (PMC5386982; doi:10.3389/fpls.2017.00476)
Supplement: Table S5 — Summary of the most important SNPs as cofactors related to different QTLs in the “98–99” parent for 2015 and 2016. [file Table5.DOCX]

**Table S5** Summary of the most important SNPs as cofactors related to different QTLs in the ‘98-99’ parent for 2015 and 2016.

| **Year** | **Trait** | **Set of cofactors** | **Map (cM)** | **ln-likelihood** | **var** | **Expl (%)** | **p** | **LOD_α=0.05_** | **LOD** | **K* (df)** | **class 1: mean** | **class 2: mean** | **class 3: mean** |
| --- | --- | --- | --- | --- | --- | --- | --- | --- | --- | --- | --- | --- | --- |
| 2015 | RT | set of 1 cofactor |  | -250.043 | 297.961 | 46.3 |  |  |  |  |  |  |  |
|  |  | S4_11357872 | 43.2 | -265.534 | 554.726 | 0.0 | 0.000 | 3.20 | 6.73 | 8.391** | hh : 206.312 | hk : 223.438 | kk : 235.400 |
|  | I_AD__1-2 | set of 1 cofactor |  | 181.521 | 0.02949 | 31.5 |  |  |  |  |  |  |  |
|  |  | S3_4166803 | 21.4 | 813.674 | 0.04307 | 0.0 | 0.000 | 3.30 | 4.35 | 11.774**** | hh : 0.196 | hk : 0.338 | kk : 0.466 |
|  | SKC | set of 3 cofactors |  | -497.812 | 0.18331 | 75.8 |  |  |  |  |  |  |  |
|  |  | S3_13627046 | 46.2 | -587.603 | 0.43233 | 42.9 | 0.000 | 3.30 | 6.49 | 4.153** | ll : 3.218 | lm : 3.615 |  |
|  |  | S3_13878544 | 47.9 | -584.408 | 0.43339 | 42.7 | 0.001 | 3.30 | 5.92 | 4.435** | ll : 3.200 | lm : 3.607 |  |
|  |  | S3_14698248 | 49.7 | -589.982 | 0.44389 | 41.3 | 0.000 | 3.30 | 6.61 | 5.214** | ll : 3.629 | lm : 3.193 |  |
|  |  | set of 1 cofactor |  | -591.381 | 0.41781 | 44.8 |  |  |  |  |  |  |  |
|  |  | S4_10872195 | 42.1 | -742.074 | 0.75654 | 0.0 | 0.000 | 3.20 | 6.54 | 12.240****** | ll : 3.757 | lm : 2.916 |  |
|  | SSC_2 | set of 1 cofactor |  | -126.025 | 622.955 | 27.1 |  |  |  |  |  |  |  |
|  |  | S1_19630503 | 63.6 | -134.534 | 854.079 | 0.0 | 0.001 | 3.40 | 3.70 | 16.313******* | ll : 15.467 | lm : 18.500 |  |
| 2016 | RT | set of 1 cofactor |  | -217.887 | 142.035 | 49.6 |  |  |  |  |  |  |  |
|  |  | S4_12564956 | 47.2 | -233.167 | 281.724 | 0.0 | 0.000 | 3.20 | 6.64 | 6.928** | hh : 208.462 | hk : 215.067 | kk : 227.167 |
|  | SKC | set of 2 cofactors |  | -560.586 | 0.44954 | 52.2 |  |  |  |  |  |  |  |
|  |  | S3_10446158 | 41.7 | -645.405 | 0.61132 | 35.0 | 0.001 | 3.00 | 2.77 | 10.672**** | hh : 4.100 | hk : 3.533 | kk: 2.933 |
|  |  | S3_13221856 | 45.6 | -699.585 | 0.74168 | 21.1 | 0.000 | 3.00 | 5.12 | 16.438****** | hh : 3.214 | hk : 3.178 | kk : 4.384 |
|  |  | set of 3 cofactors |  | -556.878 | 0.36017 | 61.7 |  |  |  |  |  |  |  |
|  |  | S4_8059311 | 34.4 | -615.982 | 0.47324 | 49.7 | 0.008 | 3.30 | 5.46 | 9.077**** | ll : 3.818 | lm : 2.952 |  |
|  |  | S4_9335602 | 37.4 | -622.140 | 0.53994 | 42.6 | 0.005 | 3.30 | 4.23 | 7.787*** | ll : 3.729 | lm : 2.944 |  |
|  |  | S4_10872195 | 42.1 | -608.703 | 0.49381 | 47.5 | 0.016 | 3.30 | 5.56 | 10.068**** | ll : 32.6 3.843 | lm : 2.954 |  |

Ripening time (RT), chlorophyll index (I_AD_), skin color (SKC), soluble solids content (SSC). All the traits were evaluated at the harvest date, while I_AD_, firmness and soluble solids were evaluated at two maturity states, at the harvest date (_1) and one week after harvest (_2). K*: *0.1, **0.05***0.01, ****0.005, *****0.001, ******0.0005, *******0.0001.
