# Supplementary material for: Genotyping by Sequencing for SNP-Based Linkage Analysis and Identification of QTLs Linked to Fruit Quality Traits in Japanese Plum (Prunus salicina Lindl.)
Source: Front Plant Sci. 2017 Apr 11;8:476. doi: 10.3389/fpls.2017.00476 (PMC5386982; doi:10.3389/fpls.2017.00476)
Supplement: Table S6 — Summary of the most important SNPs as cofactors related to different QTLs in the “Angeleno” parent for 2015 and 2016. [file Table6.DOCX]

**Table S6** Summary of the most important SNPs as cofactors related to different QTLs in the ‘Angeleno’ parent for 2015 and 2016.

| **Year** | **Trait** | **Set of cofactors** | **Map (cM)** | **ln-likelihood** | **var** | **Expl (%)** | **p** | **LOD_α=0.05_** | **LOD** | **K* (df)** | **class 1 (bp): mean** | **class 2 (bp): mean** | **class 3 (bp): mean** |
| --- | --- | --- | --- | --- | --- | --- | --- | --- | --- | --- | --- | --- | --- |
| 2015 | RT | set of 1 cofactor |  | -248.550 | 308.736 | 44.3 |  |  |  |  |  |  |  |
|  |  | S4_11967712 | 38.8 | -265.534 | 554.726 | 0.0 | 0.000 | 3.70 | 7.38 | 19.522******* | nn : 242.526 | np : 210.179 |  |
|  | I_AD__1-2 | set of 1 cofactor |  | 199.630 | 0.02608 | 39.4 |  |  |  |  |  |  |  |
|  |  | S3_8549572 | 32.1 | 813.674 | 0.04307 | 0.0 | 0.000 | 3.00 | 5.14 | 15.080****** | nn : 0.437 | np : 0.215 |  |
|  | SKC | set of 2 cofactors |  | -462.715 | 0.19485 | 74.2 |  |  |  |  |  |  |  |
|  |  | S3_13359114 | 53.8 | -601.366 | 0.43976 | 41.9 | 0.000 | 2.80 | 7.07 | 23.427******* | hh : 4.384 | hk : 3.068 | kk : 3.187 |
|  |  | S3_13688816 | 59.6 | -579.247 | 0.41808 | 44.7 | 0.000 | 2.80 | 6.11 | 10.043**** | nn : 3.037 | np : 3.709 |  |
|  |  | set of 1 cofactor |  | -623.361 | 0.48483 | 35.9 |  |  |  |  |  |  |  |
|  |  | S4_9700717 | 31.1 | -742.074 | 0.75654 | 0.0 | 0.000 | 3.30 | 5.16 | 7.927**** | nn : 2.900 | np : 3.657 |  |
|  | SSC_1 | set of 1 cofactor |  | -133.428 | 583.033 | 28.7 |  |  |  |  |  |  |  |
|  |  | S6_23276829 | 57.0 | -143.221 | 817.254 | 0.0 | 0.000 | 3.20 | 4.25 | 15.658****** | hh : 16.403 | hk : 18.422 | kk : 14.490 |
| 2016 | RT | set of 5 cofactors |  | -191.612 | 407.058 | 85.6 |  |  |  |  |  |  |  |
|  |  | S4_9765977 | 31.2 | -196.749 | 622.729 | 77.9 | 0.016 | 3.60 | 5.33 | 18.197******* | nn : 227.526 | np : 208.871 |  |
|  |  | S4_10329477 | 32.4 | -201.588 | 758.197 | 73.1 | 0.000 | 3.60 | 6.01 | 8.159** | hh : 224.900 | hk : 217.867 | kk : 206.867 |
|  |  | S4_11357872 | 36.1 | -197.351 | 671.176 | 76.2 | 0.009 | 3.60 | 8.45 | 8.397** | hh : 206.867 | hk : 217.533 | kk : 225.900 |
|  |  | S4_11643825 | 38.3 | -203.345 | 762.326 | 72.9 | 0.000 | 3.60 | 4.29 | 13.352****** | nn : 227.850 | np : 209.457 |  |
|  |  | S4_11967712 | 38.8 | -198.270 | 425.261 | 84.9 | 0.004 | 3.60 | 7.15 | 21.592******* | nn : 231.333 | np : 208.757 |  |
|  | FW | set of 1 cofactor |  | -198.068 | 778.447 | 39.0 |  |  |  |  |  |  |  |
|  |  | S7_20598519 | 58.4 | -211.391 | 127.623 | 0.0 | 0.000 | 3 | 5.79 | 17.195******* | nn : 51.951 | np : 38.236 |  |
|  | SHP | set of 5 cofactors |  | -492.707 | 0.11164 | 85.0 |  |  |  |  |  |  |  |
|  |  | S7_16271600 | 46.0 | -575.219 | 0.43149 | 42.1 | 0.001 | 3.30 | 3.91 | 15.412******* | nn : 3.354 | np : 2.500 |  |
|  |  | S7_17669210 | 51.1 | -570.963 | 0.41163 | 44.8 | 0.001 | 3.30 | 3.88 | 15.033****** | nn : 3.333 | np : 2.454 |  |
|  |  | S7_19957224 | 57.8 | -576.313 | 0.45442 | 39.0 | 0.001 | 3.30 | 4.29 | 14.796****** | nn : 3.314 | np : 2.368 |  |
|  |  | S7_20797039 | 61.8 | -568.191 | 0.39894 | 46.5 | 0.002 | 3.30 | 4.72 | 14.785****** | nn : 3.305 | np : 2.333 |  |
|  |  | S7_20956328 | 61.9 | -568.313 | 0.40127 | 46.1 | 0.002 | 3.30 | 4.73 | 14.782****** | nn : 2.368 | np : 3.305 |  |
|  | SKC | set of 3 cofactors |  | -418.248 | 0.13607 | 85.5 |  |  |  |  |  |  |  |
|  |  | S3_12879559 | 52.3 | -468.189 | 0.13835 | 85.3 | 0.019 | 3.10 | 8.15 | 25.812******* | nn : 2.857 | np : 4.111 |  |
|  |  | S3_13359114 | 53.8 | -468.954 | 0.13655 | 85.5 | 0.017 | 3.10 | 7.9 | 20.434******* | hh : 4.500 | hk : 3.250 | kk : 3.066 |
|  |  | S3_14250289 | 61.1 | -535.217 | 0.39512 | 58.0 | 0.000 | 3.10 | 4.55 | 14.682****** | nn : 3.000 | np : 3.896 |  |
|  |  | set of 1 cofactor |  | -624.328 | 0.56320 | 40.1 |  |  |  |  |  |  |  |
|  |  | S4_11967712 | 38.8 | -763.449 | 0.94016 | 0.0 | 0.000 | 3.30 | 6.04 | 6.169** | nn : 2.944 | np : 3.729 |  |
|  | OVC | set of 1 cofactor |  | -491.889 | 0.34963 | 48.9 |  |  |  |  |  |  |  |
|  |  | S4_11967712 | 38.8 | -676.215 | 0.68460 | 0.0 | 0.000 | 3.9 | 8.01 | 16.584******* | nn : 2.730 | np : 3.781 |  |

Ripening time (RT), chlorophyll index (I_AD_), skin color (SKC), soluble solids content (SSC), over color (OVC). All the traits were evaluated at the harvest date, while I_AD_, firmness and soluble solids were evaluated at two maturity states, at the harvest date (_1) and one week after harvest (_2). K*: *0.1, **0.05***0.01, ****0.005, *****0.001, ******0.0005, *******0.0001.
