# Supplementary material for: Genotyping by Sequencing for SNP-Based Linkage Analysis and Identification of QTLs Linked to Fruit Quality Traits in Japanese Plum (Prunus salicina Lindl.)
Source: Front Plant Sci. 2017 Apr 11;8:476. doi: 10.3389/fpls.2017.00476 (PMC5386982; doi:10.3389/fpls.2017.00476)
Supplement: Table S7 — Marker trait association by Mixed Linear Model (MLM) by TASSEL v5 in 2015. [file Table7.docx]

**Table S7.** Marker trait association by Mixed Linear Model (MLM) by TASSEL v5 in 2015.

| **Trait** | **Marker** | **Chr** | **Pos** | **df** | **F** | **p** | **Error df** | **R^2^** |
| --- | --- | --- | --- | --- | --- | --- | --- | --- |
| RT | S4_8917404 | 4 | 8917404 | 2 | 5.380 | 0.008 | 49 | 0.200 |
| RT | S4_8917414 | 4 | 8917414 | 2 | 5.380 | 0.008 | 49 | 0.200 |
| RT | S4_9101443 | 4 | 9101443 | 2 | 6.005 | 0.005 | 47 | 0.279 |
| RT | S4_9218570 | 4 | 9218570 | 2 | 6.397 | 0.003 | 58 | 0.227 |
| RT | S4_9680253 | 4 | 9680253 | 2 | 5.892 | 0.005 | 57 | 0.209 |
| RT | S4_9680254 | 4 | 9680254 | 2 | 5.892 | 0.005 | 57 | 0.209 |
| RT | S4_9680317 | 4 | 9680317 | 2 | 5.671 | 0.006 | 57 | 0.204 |
| RT | S4_9680815 | 4 | 9680815 | 2 | 6.055 | 0.004 | 56 | 0.219 |
| RT | S4_9701110 | 4 | 9701110 | 2 | 7.736 | 0.001 | 47 | 0.359 |
| RT | S4_9701133 | 4 | 9701133 | 2 | 7.736 | 0.001 | 47 | 0.359 |
| RT | S4_10385736 | 4 | 10385736 | 2 | 8.280 | 0.001 | 52 | 0.343 |
| RT | S4_10732809 | 4 | 10732809 | 2 | 5.954 | 0.005 | 53 | 0.256 |
| RT | S4_11298897 | 4 | 11298897 | 2 | 6.695 | 0.003 | 47 | 0.290 |
| RT | S4_11966772 | 4 | 11966772 | 2 | 5.545 | 0.007 | 51 | 0.223 |
| I_AD__1-2 | S3_6347850 | 3 | 6347850 | 1 | 10.991 | 0.002 | 57 | 0.200 |
| I_AD__1-2 | S3_9899262 | 3 | 9899262 | 2 | 6.450 | 0.003 | 58 | 0.234 |
| I_AD__1-2 | S3_9899299 | 3 | 9899299 | 2 | 6.450 | 0.003 | 58 | 0.234 |
| I_AD__1-2 | S3_9899311 | 3 | 9899311 | 2 | 6.450 | 0.003 | 58 | 0.234 |
| I_AD__1-2 | S3_10620359 | 3 | 10620359 | 2 | 7.974 | 0.001 | 58 | 0.289 |
| I_AD__1-2 | S3_13023332 | 3 | 13023332 | 1 | 12.748 | 0.001 | 56 | 0.232 |
| I_AD__1-2 | S3_14797997 | 3 | 14797997 | 1 | 12.846 | 0.001 | 55 | 0.237 |
| I_AD__1-2 | S3_14798747 | 3 | 14798747 | 2 | 9.451 | 0.000 | 56 | 0.345 |
| I_AD__1-2 | S3_15037940 | 3 | 15037940 | 1 | 15.947 | 0.000 | 58 | 0.289 |
| SKC | S3_12589005 | 3 | 12589005 | 2 | 8.071 | 0.001 | 58 | 0.276 |
| SKC | S3_13357602 | 3 | 13357602 | 2 | 8.455 | 0.001 | 54 | 0.297 |
| SKC | S3_13357607 | 3 | 13357607 | 2 | 8.455 | 0.001 | 54 | 0.297 |
| SKC | S3_13357608 | 3 | 13357608 | 2 | 8.455 | 0.001 | 54 | 0.297 |
| SKC | S3_13633241 | 3 | 13633241 | 2 | 8.129 | 0.001 | 58 | 0.278 |
| SKC | S3_13743683 | 3 | 13743683 | 2 | 8.756 | 0.001 | 47 | 0.393 |
| SKC | S3_14537793 | 3 | 14537793 | 2 | 9.136 | 0.000 | 58 | 0.313 |
| SKC | S3_15004198 | 3 | 15004198 | 2 | 6.991 | 0.002 | 57 | 0.265 |
