# Supplementary material for: Genotyping by Sequencing for SNP-Based Linkage Analysis and Identification of QTLs Linked to Fruit Quality Traits in Japanese Plum (Prunus salicina Lindl.)
Source: Front Plant Sci. 2017 Apr 11;8:476. doi: 10.3389/fpls.2017.00476 (PMC5386982; doi:10.3389/fpls.2017.00476)
Supplement: Table S8 — Marker trait association by Mixed Linear Model (MLM) by TASSEL v5 in 2016. [file Table8.docx]

**Table S8.** Marker trait association by Mixed Linear Model (MLM) by TASSEL v5 in 2016.

| **Trait** | **Marker** | **Chr** | **Pos** | **df** | **F** | **p** | **Error df** | **R^2^** |
| --- | --- | --- | --- | --- | --- | --- | --- | --- |
| RT | S4_8917404 | 4 | 8917404 | 2 | 5.768 | 0.006 | 46 | 0.225 |
| RT | S4_8917414 | 4 | 8917414 | 2 | 5.768 | 0.006 | 46 | 0.225 |
| RT | S4_9006214 | 4 | 9006214 | 2 | 5.489 | 0.008 | 45 | 0.225 |
| RT | S4_9101443 | 4 | 9101443 | 2 | 5.731 | 0.007 | 44 | 0.239 |
| RT | S4_9584460 | 4 | 9584460 | 2 | 5.956 | 0.005 | 55 | 0.217 |
| RT | S4_9584471 | 4 | 9584471 | 2 | 5.956 | 0.005 | 55 | 0.217 |
| RT | S4_9680253 | 4 | 9680253 | 2 | 7.531 | 0.001 | 54 | 0.276 |
| RT | S4_9680254 | 4 | 9680254 | 2 | 7.531 | 0.001 | 54 | 0.276 |
| RT | S4_9680317 | 4 | 9680317 | 2 | 5.740 | 0.006 | 54 | 0.210 |
| RT | S4_9680815 | 4 | 9680815 | 2 | 6.950 | 0.002 | 53 | 0.264 |
| RT | S4_9701110 | 4 | 9701110 | 2 | 8.091 | 0.001 | 44 | 0.338 |
| RT | S4_9701133 | 4 | 9701133 | 2 | 8.091 | 0.001 | 44 | 0.338 |
| RT | S4_9755136 | 4 | 9755136 | 2 | 6.394 | 0.004 | 51 | 0.248 |
| RT | S4_9755292 | 4 | 9755292 | 2 | 11.173 | 0.000 | 51 | 0.425 |
| RT | S4_10626589 | 4 | 10626589 | 2 | 10.430 | 0.000 | 54 | 0.387 |
| RT | S4_10877413 | 4 | 10877413 | 1 | 18.230 | 0.000 | 55 | 0.333 |
| RT | S4_11033151 | 4 | 11033151 | 1 | 18.230 | 0.000 | 55 | 0.333 |
| RT | S4_11128757 | 4 | 11128757 | 1 | 18.230 | 0.000 | 55 | 0.333 |
| RT | S4_11207166 | 4 | 11207166 | 1 | 18.230 | 0.000 | 55 | 0.333 |
| RT | S4_11233844 | 4 | 11233844 | 1 | 18.230 | 0.000 | 55 | 0.333 |
| RT | S4_11289165 | 4 | 11289165 | 1 | 18.230 | 0.000 | 55 | 0.333 |
| RT | S4_11357817 | 4 | 11357817 | 1 | 18.230 | 0.000 | 55 | 0.333 |
| RT | S4_11367504 | 4 | 11367504 | 1 | 16.304 | 0.000 | 55 | 0.298 |
| RT | S4_11620023 | 4 | 11620023 | 2 | 8.499 | 0.001 | 55 | 0.310 |
| RT | S4_11620051 | 4 | 11620051 | 2 | 8.499 | 0.001 | 55 | 0.310 |
| RT | S4_11967712 | 4 | 11967712 | 1 | 16.304 | 0.000 | 55 | 0.298 |
| FW | S7_16560305 | 7 | 16560305 | 2 | 5.764 | 0.007 | 43 | 0.276 |
| FW | S7_16560307 | 7 | 16560307 | 2 | 5.764 | 0.007 | 43 | 0.276 |
| FW | S7_16560327 | 7 | 16560327 | 2 | 5.764 | 0.007 | 43 | 0.276 |
| FW | S7_17123208 | 7 | 17123208 | 2 | 6.620 | 0.003 | 45 | 0.285 |
| FW | S7_20394853 | 7 | 20394853 | 2 | 5.740 | 0.006 | 48 | 0.254 |
| FW | S7_20627708 | 7 | 20627708 | 2 | 10.629 | 0.000 | 48 | 0.432 |
| FW | S7_20857443 | 7 | 20857443 | 2 | 7.384 | 0.002 | 54 | 0.271 |
| SKC | S3_12589005 | 3 | 12589005 | 2 | 8.527 | 0.001 | 55 | 0.325 |
| SKC | S3_12649421 | 3 | 12649421 | 2 | 8.073 | 0.001 | 53 | 0.317 |
| SKC | S3_12670263 | 3 | 12670263 | 1 | 17.648 | 0.000 | 55 | 0.336 |
| SKC | S3_12788048 | 3 | 12788048 | 1 | 16.417 | 0.000 | 55 | 0.312 |
| SKC | S3_12879559 | 3 | 12879559 | 1 | 16.278 | 0.000 | 55 | 0.310 |
| SKC | S3_13148131 | 3 | 13148131 | 1 | 16.417 | 0.000 | 55 | 0.312 |
| SKC | S3_13169525 | 3 | 13169525 | 1 | 15.186 | 0.000 | 54 | 0.305 |
| SKC | S3_13169526 | 3 | 13169526 | 1 | 15.186 | 0.000 | 54 | 0.305 |
| SKC | S3_13169527 | 3 | 13169527 | 1 | 15.186 | 0.000 | 54 | 0.305 |
| SKC | S3_13743448 | 3 | 13743448 | 2 | 7.804 | 0.001 | 49 | 0.319 |
| SKC | S3_13766436 | 3 | 13766436 | 1 | 18.576 | 0.001 | 55 | 0.354 |
