# Supplementary material for: Genotyping by Sequencing for SNP-Based Linkage Analysis and Identification of QTLs Linked to Fruit Quality Traits in Japanese Plum (Prunus salicina Lindl.)
Source: Front Plant Sci. 2017 Apr 11;8:476. doi: 10.3389/fpls.2017.00476 (PMC5386982; doi:10.3389/fpls.2017.00476)
Supplement: Table S9 — Marker trait association by Mixed Linear Model (MLM) by TASSEL v5 for both years. [file Table9.docx]

**Table S9** Marker trait association by Mixed Linear Model (MLM) by TASSEL v5 for both years.

| **Trait** | **Marker** | **Chr** | **Pos** | **df** | **F** | **p** | **Error df** | **R^2^** |
| --- | --- | --- | --- | --- | --- | --- | --- | --- |
| RT | S4_8917404 | 4 | 8917404 | 2 | 6.532 | 0.003 | 46 | 0.254 |
| RT | S4_8917414 | 4 | 8917414 | 2 | 6.532 | 0.003 | 46 | 0.254 |
| RT | S4_9101443 | 4 | 9101443 | 2 | 6.318 | 0.004 | 44 | 0.285 |
| RT | S4_9218570 | 4 | 9218570 | 2 | 7.539 | 0.001 | 55 | 0.281 |
| RT | S4_9680253 | 4 | 9680253 | 2 | 8.378 | 0.001 | 54 | 0.314 |
| RT | S4_9680254 | 4 | 9680254 | 2 | 8.378 | 0.001 | 54 | 0.314 |
| RT | S4_9680317 | 4 | 9680317 | 2 | 6.379 | 0.003 | 54 | 0.240 |
| RT | S4_9680815 | 4 | 9680815 | 2 | 7.125 | 0.002 | 53 | 0.274 |
| RT | S4_9701110 | 4 | 9701110 | 2 | 8.689 | 0.001 | 44 | 0.391 |
| RT | S4_9701133 | 4 | 9701133 | 2 | 8.689 | 0.001 | 44 | 0.391 |
| RT | S4_10385736 | 4 | 10385736 | 2 | 6.638 | 0.003 | 49 | 0.278 |
| RT | S4_10732809 | 4 | 10732809 | 2 | 6.135 | 0.004 | 50 | 0.270 |
| RT | S4_11298897 | 4 | 11298897 | 2 | 7.029 | 0.002 | 44 | 0.311 |
| RT | S4_11357817 | 4 | 11357817 | 1 | 12.640 | 0.001 | 55 | 0.236 |
| RT | S4_11367504 | 4 | 11367504 | 1 | 11.977 | 0.001 | 55 | 0.223 |
| RT | S4_11620023 | 4 | 11620023 | 2 | 6.079 | 0.004 | 55 | 0.227 |
| RT | S4_11620051 | 4 | 11620051 | 2 | 6.079 | 0.004 | 55 | 0.227 |
| RT | S4_11967712 | 4 | 11967712 | 1 | 11.977 | 0.001 | 55 | 0.223 |
| RT | S4_11970178 | 4 | 11970178 | 2 | 6.030 | 0.005 | 53 | 0.228 |
| SKC | S3_12589005 | 3 | 12589005 | 2 | 8.948 | 0.000 | 55 | 0.340 |
| SKC | S3_12649421 | 3 | 12649421 | 2 | 7.399 | 0.002 | 53 | 0.289 |
| SKC | S3_12670263 | 3 | 12670263 | 1 | 17.808 | 0.000 | 55 | 0.339 |
| SKC | S3_12788048 | 3 | 12788048 | 1 | 16.459 | 0.000 | 55 | 0.313 |
| SKC | S3_12879559 | 3 | 12879559 | 1 | 15.978 | 0.000 | 55 | 0.304 |
| SKC | S3_13148131 | 3 | 13148131 | 1 | 16.459 | 0.000 | 55 | 0.313 |
| SKC | S3_13169525 | 3 | 13169525 | 1 | 15.515 | 0.000 | 54 | 0.306 |
| SKC | S3_13169526 | 3 | 13169526 | 1 | 15.515 | 0.000 | 54 | 0.306 |
| SKC | S3_13169527 | 3 | 13169527 | 1 | 15.515 | 0.000 | 54 | 0.306 |
| SKC | S3_13633241 | 3 | 13633241 | 2 | 8.574 | 0.001 | 55 | 0.326 |
| SKC | S3_13672061 | 3 | 13672061 | 2 | 7.841 | 0.001 | 53 | 0.294 |
| SKC | S3_13677777 | 3 | 13677777 | 2 | 7.657 | 0.001 | 52 | 0.306 |
| SKC | S3_13712820 | 3 | 13712820 | 2 | 7.525 | 0.001 | 55 | 0.286 |
| SKC | S3_13712821 | 3 | 13712821 | 2 | 7.525 | 0.001 | 55 | 0.286 |
| SKC | S3_13712843 | 3 | 13712843 | 2 | 7.525 | 0.001 | 55 | 0.286 |
| SKC | S3_13743448 | 3 | 13743448 | 2 | 7.731 | 0.001 | 49 | 0.311 |
| SKC | S3_13766436 | 3 | 13766436 | 1 | 16.423 | 0.000 | 55 | 0.312 |
| FW | S7_16948180 | 7 | 16948180 | 2 | 6.306 | 0.004 | 55 | 0.234 |
| FW | S7_16948196 | 7 | 16948196 | 2 | 6.306 | 0.004 | 55 | 0.234 |
| FW | S7_17123208 | 7 | 17123208 | 2 | 6.844 | 0.003 | 45 | 0.294 |
| FW | S7_18353796 | 7 | 18353796 | 2 | 7.197 | 0.002 | 50 | 0.275 |
| FW | S7_19582925 | 7 | 19582925 | 2 | 5.579 | 0.007 | 50 | 0.237 |
| FW | S7_19582927 | 7 | 19582927 | 2 | 5.579 | 0.007 | 50 | 0.237 |
| FW | S7_19657181 | 7 | 19657181 | 2 | 6.191 | 0.004 | 52 | 0.241 |
| FW | S7_19657200 | 7 | 19657200 | 2 | 6.513 | 0.003 | 52 | 0.253 |
| FW | S7_19769827 | 7 | 19769827 | 2 | 6.233 | 0.004 | 54 | 0.240 |
| FW | S7_20099896 | 7 | 20099896 | 1 | 14.273 | 0.000 | 55 | 0.264 |
| FW | S7_20394853 | 7 | 20394853 | 2 | 6.652 | 0.003 | 48 | 0.281 |
| FW | S7_20501952 | 7 | 20501952 | 2 | 7.032 | 0.002 | 55 | 0.261 |
| FW | S7_20627708 | 7 | 20627708 | 2 | 9.721 | 0.000 | 48 | 0.421 |
| FW | S7_20735408 | 7 | 20735408 | 2 | 5.253 | 0.009 | 50 | 0.231 |
| FW | S7_20840252 | 7 | 20840252 | 2 | 6.042 | 0.004 | 55 | 0.224 |
| FW | S7_20857443 | 7 | 20857443 | 2 | 8.077 | 0.001 | 54 | 0.302 |
| FW | S7_20857519 | 7 | 20857519 | 2 | 6.942 | 0.002 | 55 | 0.257 |
