# Supplementary material for: Genotyping by Sequencing for SNP-Based Linkage Analysis and Identification of QTLs Linked to Fruit Quality Traits in Japanese Plum (Prunus salicina Lindl.)
Source: Front Plant Sci. 2017 Apr 11;8:476. doi: 10.3389/fpls.2017.00476 (PMC5386982; doi:10.3389/fpls.2017.00476)
Supplement: Table S10 — Summary of nearest genes related to the most important QTLs identified (ripening time, RT; fruit weight, FW; and skin color, SKC). [file Table10.DOCX]

**Table S10.** Summary of nearest genes related to the most important QTLs identified (ripening time, RT; fruit weight, FW and skin color, SKC).

| Trait | Locus | Ubication | Distance | Gene | Protein | Name |
| --- | --- | --- | --- | --- | --- | --- |
| RT | S4_9700717 | 9700021..9703458 | 1 bp | ppa002367m | XP_007211362.1 | TRX family/TPR 1/TPR 11/TPR17/TPR 19 |
| RT | S4_9765977 | 9765004..9768326 | 1 bp | ppa006643m | XP_007213851.1 | Med15 |
| RT | S4_10329477 | 10329524..10330247 | 200 bp | ppa011129m | XP_007212003.1 | AdoMet Mtases |
| RT | S4_11357872 | 11357222..11358821 | 1 bp | ppa009987m | XP_007211811.1 | PLN00024 |
| RT | S4_11643825 | 11641197..11644509 | 1 bp | ppa018380m | XP_007214138.1 | B lectin/PAN AP-Plant/STKc IRAK/Pkinase/S-locus glycop/B-lectin |
| RT | S4_11967712 | 11964322..11968405 | 1 bp | ppa024127m | XP_007214693.1 | STYKc/STKc IRAK/S-locus glycop/PAN 2 |
| RT | S4_12564956 | 12556502..12565337 | 1 bp | ppa000393m | XP_007214561.1 | Chorein-N |
| FW | S7_16271600 | [16273556..16277759](http://services.appliedgenomics.org/fgb2/iga/prunus_public/gbrowse/prunus_public?name=scaffold_7:16273556..16277759) | 10 kbp | ppa002163m | XP_007204625.1 | STYKc/PKc like |
| FW | S7_17669210 | [17663214..17668753](http://services.appliedgenomics.org/fgb2/iga/prunus_public/gbrowse/prunus_public?name=scaffold_7:17663214..17668753) | 1 kbp | ppa000366m | XP_007201926.1 | HATPase c/HisKA/PRK11107/REC |
| FW | S7_19957224 | 19955832..19957718 | 1 bp | ppa008804m | XP_007203887.1 | MIOX |
| FW | S7_20598519 | 20597996..20600733 | 1 bp | ppa003799m | XP_007204566.1 | Caudal activation/Frigida |
| FW | S7_20797039 | [20798037..20802680](http://services.appliedgenomics.org/fgb2/iga/prunus_public/gbrowse/prunus_public?name=scaffold_7:20798037..20802680) | 2 kbp | ppa004615m | XP_007204856.1 | MFS/Sugar tr |
| FW | S7_20956328 | 20956002..20961542 | 1 bp | ppa001241m | XP_007204288.1 | PLN03192/CAP ED/ANK/Ion trans 2/KHA/ANK |
| SKC | S3_12879559 | 12877574..12879431 | 1 kbp | ppa026640m | XP_007216530.1 | SANT/Myb DNA-binding |
| SKC | S3_13221856 | 13222842..13225996 | 5 kbp | ppa010028m | XP_007217261.1 | Gem1/ATP bind-1 |
| SKC | S3_13359114 | 13358912..13363070 | 1 bp | ppa002769m | XP_007217629.1 | ACT-3PGDH/PRK13581/PGDH-4 |
| SKC | S3_13627046 | 13631252..13633463 | 10 kbp | ppa002417m | XP_007215006.1 | PLN02778/dTDP GD SDR e/PLN02260 |
| SKC | S3_13688816 | 13687689..13688818 | 1 bp | ppa013920m | XP_007215186.1 | Function unknown |
| SKC | S3_13878544 | 13875504..13880408 | 1 bp | ppa001438m | XP_007214963.1 | COG5 |
| SKC | S3_14250289 | 14248419..14251355 | 1 bp | ppa005584m | XP_007215363.1 | PLN02268/NAD-binding-8 |
| SKC | S3_14698248 | 14697108..14702552 | 1 bp | ppa003405m | XP_007215027.2 | p450 |
| SKC | S4_8059311 | [8063832..8064757](http://services.appliedgenomics.org/fgb2/iga/prunus_public/gbrowse/prunus_public?name=scaffold_4:8063832..8064757) | 20 kbp | ppa020275m | XP_007213044.1 | aeEF2 snRNP like-IV/aeEF2 snRNP like-C/EFG III-like |
| SKC | S4_9335602 | 9333656..9335710 | 1 bp | ppa023386m | XP_007213481.1 | STYKc/PKc like |
| SKC | S4_9700717 | 9700021..9703458 | 1 bp | ppa002367m | XP_007211362.1 | TRX family/TPR 1/TPR 11/TPR17/TPR 19 |
| SKC | S4_10872195 | 10872510..10874220 | 100 bp | ppa019935m | XP_007212780.1 | FBD/LRR-2/F-box |
| SKC | S4_10872195 | 10872510..10874220 | 100 bp | ppa019935m | XP_007212780.1 | FBD/LRR-2/F-box |
| SKC | S4_11643825 | 11641197..11644509 | 1 bp | ppa018380m | XP_007214138.1 | B lectin/PAN AP-Plant/STKc IRAK/Pkinase/S-locus glycop/B-lectin |
| SKC | S4_11967712 | 11964322..11968405 | 1 bp | ppa024127m | XP_007214693.1 | STYKc/STKc IRAK/S-locus glycop/PAN 2 |
